# A novel STING agonist-adjuvanted pan-sarbecovirus vaccine elicits potent and durable neutralizing antibody and T cell responses in mice, rabbits and NHPs

**DOI:** 10.1038/s41422-022-00612-2

**Published:** 2022-01-19

**Authors:** Zezhong Liu, Jie Zhou, Wei Xu, Wei Deng, Yanqun Wang, Meiyu Wang, Qian Wang, Ming Hsieh, Jingming Dong, Xinling Wang, Weijin Huang, Lixiao Xing, Miaoling He, Chunlin Tao, Youhua Xie, Yilong Zhang, Youchun Wang, Jincun Zhao, Zhenghong Yuan, Chuan Qin, Shibo Jiang, Lu Lu

**Affiliations:** 1 Key Laboratory of Medical Molecular Virology (MOE/NHC/CAMS), School of Basic Medical Sciences; Shanghai Institute of Infectious Disease and Biosecurity; Biosafety Level 3 Laboratory, Shanghai Medical College, Shanghai Frontiers Science Center of Pathogenic Microbes and Infection, Fudan University, Shanghai, China; 2 Key Laboratory of Human Disease Comparative Medicine, National Health Commission of the People’s Republic of China; Beijing Key Laboratory for Animal Models of Emerging and Reemerging Infectious Diseases, Institute of Laboratory Animal Science, Chinese Academy of Medical Sciences and Comparative Medicine Center, Peking Union Medical College, Beijing, China; 3 State Key Laboratory of Respiratory Disease, National Clinical Research Center for Respiratory Disease, Guangzhou Institute of Respiratory Health, the First Affiliated Hospital of Guangzhou Medical University, Guangzhou Guangdong, China; 4 Institute of Infectious Disease, Guangzhou Eighth People’s Hospital of Guangzhou Medical University, Guangzhou Guangdong, China; 5 Guangzhou Laboratory, Bio-Island Guangzhou, China; 6 Graduate School of Peking Union Medical College, No. 9 Dongdan Santiao, Dongcheng District Beijing, China; 7 Division of HIV/AIDS and Sex-transmitted Virus Vaccines, Institute for Biological Product Control, National Institutes for Food and Drug Control (NIFDC), No. 31 Huatuo Street, Daxing District Beijing, China; 8 Fulgent Pharma LLC., 4978 Santa Anita Avenue, Temple City, CA USA

**Keywords:** Innate immunity, Immunology, Molecular biology

## Abstract

The emergence of SARS-CoV-2 variants and potentially other highly pathogenic sarbecoviruses in the future highlights the need for pan-sarbecovirus vaccines. Here, we discovered a new STING agonist, CF501, and found that CF501-adjuvanted RBD-Fc vaccine (CF501/RBD-Fc) elicited significantly stronger neutralizing antibody (nAb) and T cell responses than Alum- and cGAMP-adjuvanted RBD-Fc in mice. Vaccination of rabbits and rhesus macaques (nonhuman primates, NHPs) with CF501/RBD-Fc elicited exceptionally potent nAb responses against SARS-CoV-2 and its nine variants and 41 S-mutants, SARS-CoV and bat SARSr-CoVs. CF501/RBD-Fc-immunized hACE2-transgenic mice were almost completely protected against SARS-CoV-2 challenge, even 6 months after the initial immunization. NHPs immunized with a single dose of CF501/RBD-Fc produced high titers of nAbs. The immunized macaques also exhibited durable humoral and cellular immune responses and showed remarkably reduced viral load in the upper and lower airways upon SARS-CoV-2 challenge even at 108 days post the final immunization. Thus, CF501/RBD-Fc can be further developed as a novel pan-sarbecovirus vaccine to combat current and future outbreaks of sarbecovirus diseases.

## Introduction

Global public health is continuously threatened by emerging viruses.^1^ Coronaviruses, especially sarbecoviruses, including severe acute respiratory syndrome coronavirus 2 (SARS-CoV-2) and severe acute respiratory syndrome coronavirus (SARS-CoV), have respectively caused two global health crises within the past 20 years.^2–4^ In particular, the ongoing global pandemic of coronavirus disease of 2019 (COVID-19) caused by SARS-CoV-2 infection has resulted in more than 270 million confirmed cases and 5.3 million deaths (https://www.who.int/). The emergence of SARS-CoV-2 variants of concern (VOCs), including B.1.1.7 (Alpha),^5^ B.1.351 (Beta),^6^ P.1 (Gamma)^7^ and B.1.617.2 (Delta),^8^ and variants of interest (VOIs), such as B.1.427 (Epsilon), P.2 (Zeta), B.1.525 (Eta), B.1.526 (Iota), B.1.617.1 (Kappa), and C.37 (Lambda) (https://www.who.int/), has posed a serious challenge for the development of COVID-19 vaccines. These VOCs have shown increased transmissibility, infectivity, and resistance to humoral responses induced by some COVID-19 vaccines.^8–13^ With the worldwide spread of SARS-CoV-2 and increased immune pressure, more variants are expected to emerge in the near future. Furthermore, numerous SARS-related coronaviruses (SARSr-CoVs) identified from bats, such as WIV1, Rs3367, and RsSHC014, represent potential threats to humans since they can utilize human angiotensin-converting enzyme 2 (ACE2) as their common receptor to enter and replicate in primary airway epithelial cells.^14–16^ Similar to SARS-CoV, which infects humans through intermediate animal hosts, these bat SARSr-CoVs may also break the species barrier to infect an intermediate animal host, and then humans.^14,17,18^ Thus, the constant emergence of SARS-CoV-2 variants and the high pandemic potential of SARSr-CoVs underscore the need for the development of pan-sarbecovirus or universal coronavirus vaccines.^19^

Recent studies revealed that only one amino acid mutation, such as E484K in the spike (S) protein of SARS-CoV-2, confers resistance to the neutralization activity of convalescent or immune sera.^9^ So far, SARS-CoV-2 has shown relatively few variants. In comparison, other sarbecoviruses, such as SARS-CoV, share low homology (~76% amino acid identity for the S protein) with SARS-CoV-2,^20^ which substantially increases the difficulty of developing pan-sarbecovirus vaccines. Moreover, neutralizing antibody (nAb) titers against SARS-CoV-2 and its variants gradually decrease only a few months after vaccination with current COVID-19 vaccines.^21,22^ Therefore, how to induce potent and durable protective immunity against the currently circulating SARS-CoV-2 variants, future SARS-CoV-2 variants, and SARSr-CoVs, is now the challenge confronting vaccine developers.

Subunit vaccines are the most commonly used vaccines because of their excellent safety profiles and efficacy.^23–27^ Therefore, the development of safe and effective COVID-19 subunit vaccines is expected to play a significant role in controlling the COVID-19 pandemic. Currently, most COVID-19 subunit vaccines target mainly the spike (S) protein or receptor-binding domain (RBD). Especially, the RBD contains multiple neutralizing epitopes, and 90% neutralization was induced by the RBD in COVID-19 convalescent sera.^28–30^ Moreover, RBD-immunized mice or nonhuman primates (NHPs) exhibited protection against SARS-CoV-2 challenge.^25,31,32^ Most importantly, several RBD-binding monoclonal antibodies (mAbs), such as A23-58.1, B1-182.1, and A19-61.1 exhibit great potency against all VOCs and VOIs.^33^ Besides, S309,^34^ DH1047,^35^ BG10-19,^36^ S2X259,^37^ XG014,^38,39^ ADI55689^20^ and ADG-2,^40^ have been reported to show cross-neutralizing activity against SARS-CoV-2 and SARS-CoV, providing proof-of-concept for the development of a pan-sarbecovirus vaccine that uses RBD as an immunogen. However, we previously found that the SARS-CoV-2 RBD-Fc protein formulated with Freund’s adjuvant induced only a very limited cross-nAb response.^25^ Thus, the induction of cross-nAbs has become an obstacle hindering the development of pan-sarbecovirus vaccines.

Adjuvants are essential components of subunit vaccines by promoting potent and persistent immune responses.^41,42^ Alum (Aluminum salts), the most widely used adjuvant in humans, has had a good safety profile for many years.^43,44^ However, limited antibody immune response, poor cellular immune response, and predominantly Th2 response restrict the use of Alum as an adjuvant for many candidate antigens.^43,45^ To improve subunit vaccine efficacy, new immunostimulants able to safely and potently stimulate both humoral and cellular immune responses are constantly being sought. Among the increasing number of studies on innate immunity, stimulator of interferon genes (STING) agonist was found to be a new adjuvant by activating STING to induce the type I interferon (IFN-I) response and proinflammatory cytokine production.^46–48^ cGAMP is the most potent natural agonist of STING.^41^ We recently reported that PS-cGAMP, pulmonary surfactant–biomimetic liposome encapsulating cGAMP, could promote the induction of strong and durable heterosubtypic immunity in response to the influenza H1N1 vaccine,^49^ suggesting that a STING agonist could also be used as a COVID-19 vaccine adjuvant to induce broad- and long-lasting immune responses.

Here, we designed and synthesized several small-molecule STING agonists and found that one, CF501, showed excellent adjuvant effects. Intramuscular administration of CF501-adjuvanted SARS-CoV-2 RBD-Fc-based vaccine (CF501/RBD-Fc) could elicit significantly stronger humoral and cellular immune responses than Alum- and cGAMP-adjuvanted RBD-Fc vaccines (Alum/RBD-Fc and cGAMP/RBD-Fc) against SARS-CoV-2 and its variants, SARS-CoV and SARSr-CoVs from bats in different animal models without concomitant excess inflammation. Especially, CF501/RBD-Fc elicited extremely high titers of nAbs against the authentic SARS-CoV-2 Beta, Delta and Eta variants in NHPs, suggesting its potent neutralizing effect. In contrast, Alum/RBD-Fc induced only limited, or no cross-nAbs against SARS-CoV-2 and its variants, SARS-CoV and SARSr-CoVs. We also monitored long-term immune protection in mice and NHPs. Human ACE2 transgenic mice (hACE2-Tg mice) immunized with CF501/RBD-Fc were almost completely protected against SARS-CoV-2 challenge at 6 months post-immunization. Similarly, nAb titers in sera from CF501/RBD-Fc-immunized rhesus macaques remained at high titers for six months, and the immunized macaques were effectively protected against SARS-CoV-2 challenge, even at 108 days post-final immunization. The CF501/RBD-Fc vaccine represents a pan-sarbecovirus vaccine to combat the current circulating SARS-CoV-2 and its variants, and newly emerging SARSr-CoVs in the future. Moreover, for any future outbreak of these newly emerging viruses, CF501 might also be used as an adjuvant to boost the original protein vaccine, producing potent, broad, and long-term immune protection.

## Results

### Screening for small-molecule STING agonists as adjuvants to enhance the antibody immune response induced by the SARS-CoV-2 RBD-Fc protein in mice

**Fig. 1:**
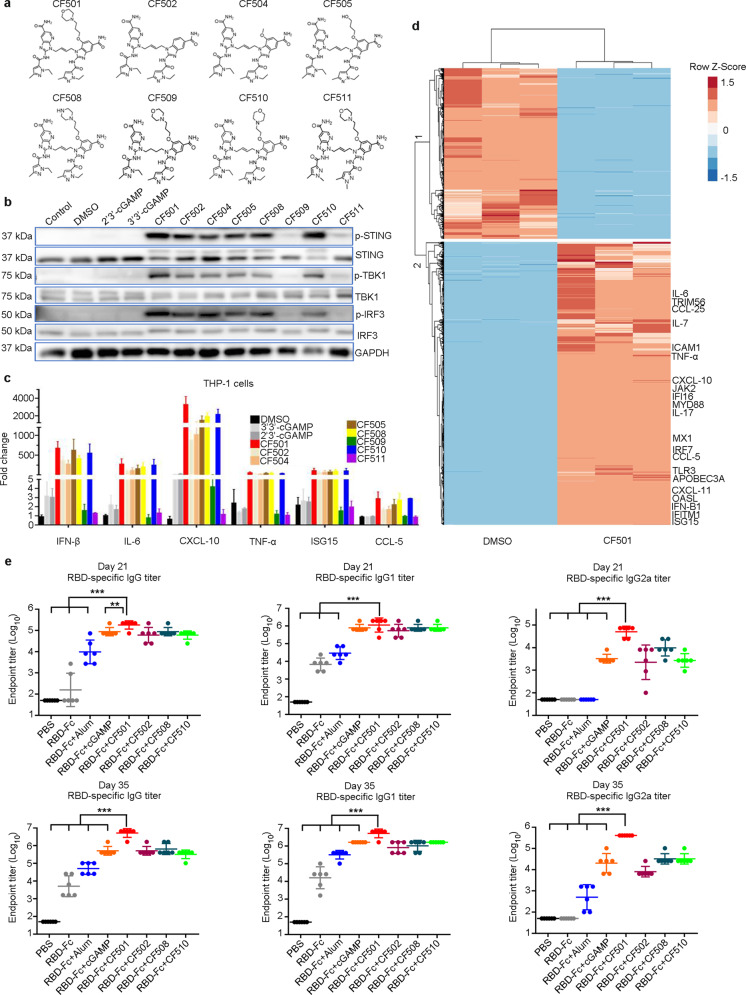
The ability of STING agonists to activate STING and enhance the humoral immune response in mice. **a** The structures of the STING agonists. **b** THP-1 cells were treated with the indicated STING agonists (10 µM) for 3 h. Cells were lysed, and immunoblotting assay was used to determine the protein expression levels. **c** Activation of IFN-β, IL-6, CXCL-10, TNF-α, ISG15 and CCL-5 following 5-h incubation with the STING agonists. Data shown are means ± SEM. **d** THP-1 cells were treated with DMSO or 10 µM CF501 for 5 h. Total RNA was extracted for RNA-seq analysis. Heatmap shows the differentially expressed genes with Log2 fold change > 2 or <–2 and an adjusted *P* value < 0.05. **e** SARS-CoV-2 RBD-specific binding IgG, IgG1, and IgG2a endpoint titers are shown at day 21 and day 35. Data shown are geometric means ± SD from six samples. Statistical analyses were performed using one-way ANOVA. *P < 0.05, **P < 0.001, ***P < 0.0001.

We designed and synthesized eight novel non-nucleotide small-molecule STING agonists (Fig. 1a; Supplementary information, Fig. S1). Activation of STING is expected to result in phosphorylation of STING, leading to downstream signaling cascades that include, i.e., the activation of TBK1 and IRF3.^46,50,51^ We found that CF501, CF502, CF504, CF505, CF508 and CF510 activated STING more effectively than cGAMP after treatment of THP-1 cells for 0.5 h (Supplementary information, Fig. S2a) or 3 h (Fig. 1b), as demonstrated by the higher level of phosphorylated STING, TBK1 and IRF3 (Fig. 1b; Supplementary information, Fig. S2a). We next measured the production of proinflammatory cytokines and type I IFNs in THP-1 cells treated with these compounds and cGAMP as a control. Consistent with the results from the activation of the STING-TBK1-IRF3 signaling pathway, THP-1 cells treated with CF501, CF502, CF504, CF505, CF508, and CF510 produced high levels of IFN-β, IL-6, CXCL-10, TNF-α, ISG-15, and CCL-5 (Fig. 1c), while only moderate or low levels of these cytokines were induced by cGAMP, CF509, and CF511 (Fig. 1c). We then selected CF501 to perform RNA-seq on THP-1 cells because of its high efficiency in activating STING. Results revealed that CF501 treatment led to rapid and robust activation of innate immune responses in THP-1 cells (Fig. 1d; Supplementary information, Fig. S2b).

Based on results from the activation of the STING signaling pathway and the efficiency of compound synthesis, CF501, CF502, CF508, and CF510 were selected to investigate whether these STING agonists could stimulate the immune system to enhance the protective immune response to a candidate COVID-19 vaccine. To accomplish this, mice were immunized intramuscularly with SARS-CoV-2 RBD-Fc dimeric protein alone or formulated with Alum, cGAMP, CF501, CF502, CF508, or CF510, respectively (Supplementary information, Fig. S2c). Mice were vaccinated three times at 14-day intervals, and the sera were collected at 21 and 35 days post-1st immunization (Supplementary information, Fig. S2c). Overall, all of these STING agonists induced stronger immune responses than Alum adjuvant (Fig. 1e; Supplementary information, Fig. S2d, e). Specifically, compared to those in the cGAMP/RBD-Fc and Alum/RBD-Fc groups, mice in the CF501/RBD-Fc group generated the strongest response to SARS-CoV-2 RBD (Fig. 1e; Supplementary information, Fig. S2d, e). Two vaccinations with CF501/RBD-Fc increased the IgG, IgG1, and IgG2a levels in mouse sera more than 1000-, 100- and 1000-fold, respectively, compared to those induced by the RBD-Fc vaccine alone (Fig. 1e). Similarly, the levels of IgG, IgG1, and IgG2a in sera from the CF501/RBD-Fc group were more than 1000-, 300-, and 8000-fold higher than those in sera from the RBD-Fc without adjuvant group after the third immunization (Fig. 1e). No detectable IgG2a in sera from mice in the Alum/RBD-Fc group was observed after the 2nd vaccination (Fig. 1e; Supplementary information, Fig. S2d), which is consistent with a previous report showing that Alum mainly induces Th2 response.^43^ In contrast, two vaccinations of the CF501/RBD-Fc elicited high titers of IgG2a, which is more than 1000-fold higher than that induced by Alum/RBD-Fc, suggesting that CF501 can strongly activate Th1 immune response (Fig. 1e; Supplementary information, Fig. S2d).

### CF501/RBD-Fc induced strong cross-nAbs against SARS-CoV-2, SARS-CoV, and SARSr-CoVs in mice

**Fig. 2:**
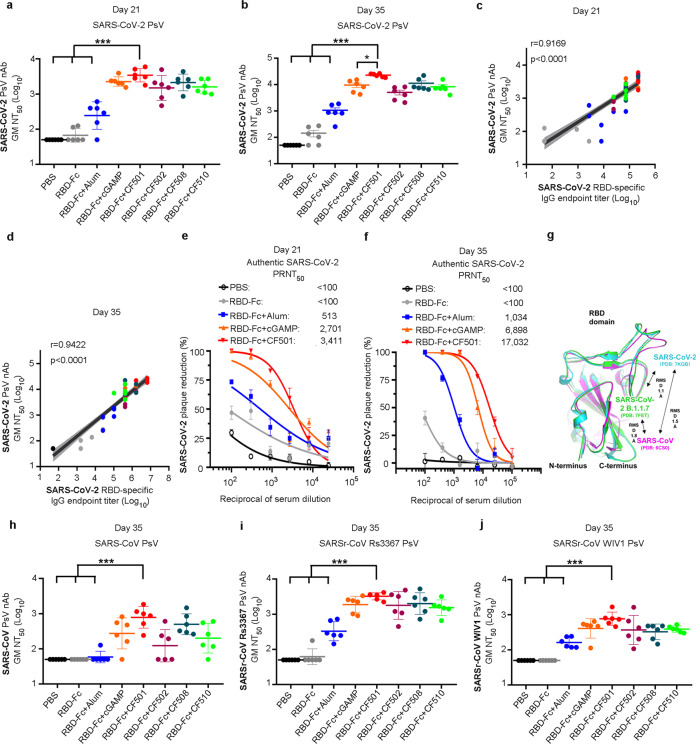
STING agonist-adjuvanted RBD-Fc induces robust cross-neutralizing antibodies against multiple sarbecoviruses in mice. **a**, **b** Neutralization activity of sera from mice immunized with RBD-Fc formulated with the indicated adjuvants against SARS-CoV-2 PsV at day 21 (**a**) and day 35 (**b**). Data are shown as geometric means ± SD from six samples. **c**, **d** Correlation between SARS-CoV-2 RBD-binding IgG endpoint titers and the NT_50_ against SARS-CoV-2 PsV in serum at day 21 (**c**) and day 35 (**d**). **e**, **f** Dose-dependent inhibitory activity of the pooled sera from indicated mouse groups against authentic SARS-CoV-2 (strain: SH01) infection at day 21 (**e**) and day 35 (**f**). Data are shown as means ± SEM. **g** Comparison of the RBD protein structures of SARS-CoV-2 (WT), SARS-CoV-2 (B.1.1.7), and SARS-CoV. **h**–**j** Cross-neutralization activity against SARS-CoV PsV (**h**), SARSr-CoV Rs3367 (**i**) and SARSr-CoV WIV1 (**j**) on day 35. Data shown are geometric means ± SD from six samples. Statistical analyses were performed using one-way ANOVA for comparison of the neutralization antibody titers. *P < 0.05, **P < 0.001, ***P < 0.0001. Spearman rank test was used to perform the correlation analysis.

To compare the nAb titers elicited by the STING agonist- and Alum-adjuvanted vaccines, we first measured the serum 50% neutralization titer (NT_50_) against SARS-CoV-2 pseudovirus (PsV) infection. Consistent with the results from binding antibody response, CF501/RBD-Fc elicited a strong serum nAb response, with a geometric mean (GM) NT_50_ 13- and 24-fold, respectively, higher than that induced by Alum/RBD-Fc at day 21 and day 35 post-1st immunization (Fig. 2a, b). CF501/RBD-Fc also elicited a significantly higher NT_50_ at day 35 post-1st immunization than did cGAMP/RBD-Fc (Fig. 2b). Furthermore, strong correlations were observed between RBD-specific IgG/IgG1/IgG2a titers and nAb titers, both at day 21 and day 35 post-1st immunization (Fig. 2c, d; Supplementary information, Fig. S3a, b).

We determined the titer of serum nAbs against authentic SARS-CoV-2. Plaque reduction assay indicated that CF501/RBD-Fc induced the most potent nAb responses with 50% plaque reduction neutralization (PRNT_50_) values of 3,411 and 17,032, respectively, after two and three immunizations, which were approximately 5.6- and 15.5-fold higher than those induced by Alum/RBD-Fc and 0.3- and 1.5-fold higher than those induced by cGAMP/RBD-Fc, respectively (Fig. 2e, f). Immunofluorescence assay further confirmed that mouse sera from the CF501/RBD-Fc group possessed strong neutralizing activity against authentic SARS-CoV-2, as demonstrated by the suppression of SARS-CoV-2 N protein expression (Supplementary information, Fig. S3c, d). Mouse sera in the CF501/RBD-Fc group also exhibited the highest 50% inhibition titers (IT_50_: 4233) against SARS-CoV-2 S-mediated cell-cell fusion, with titers about 4.8- and 2.2-fold higher than those of mice in the Alum/RBD-Fc and cGAMP/RBD-Fc groups (Supplementary information, Fig. S3e). These results suggest that the STING agonist CF501 can be used as an adjuvant to potentially enhance the vaccine’s capacity to elicit nAb responses.

SARS-CoV and some SARSr-CoVs circulating in animals pose risks for future outbreaks.^15,25^ We compared the amino acid sequences of RBD of SARS-CoV-2, SARS-CoV, and SARSr-CoVs and found that they showed 73.24%–75.59% amino acid sequence identity (Supplementary information, Fig. S3f). We also compared the RBD protein structures of SARS-CoV-2 (WT), SARS-CoV-2 (B.1.1.7), and SARS-CoV. High structure homologies of RBD proteins were found among SARS-CoV-2 (WT), SARS-CoV-2 (B.1.1.7), and SARS-CoV. These results suggest that it is possible to induce cross-neutralizing antibodies using the SARS-CoV-2 RBD protein (Fig. 2g). We, therefore, tested the cross-binding activity of antibodies to SARS-CoV RBD. Surprisingly, mouse sera from the CF501/RBD-Fc group exhibited the highest cross-binding antibody titer at day 35 (Supplementary information, Fig. S3g), which was significantly higher than those of the RBD-Fc alone, Alum/RBD-Fc and cGAMP/RBD-Fc groups. Results from the SARS-CoV PsV neutralization assay indicated that mice in the CF501/RBD-Fc group generated a robust cross-nAb response against SARS-CoV PsV with GM NT_50_ up to ~800 (Fig. 2h), which was significantly higher than the titer induced by RBD-Fc and Alum/RBD-Fc, both of which induced no detectable cross-nAbs. As expected, these antisera could not neutralize the H5N1 PsV which contains the same HIV backbone as SARS-CoV PsV (Supplementary information, Fig. S3h). We then tested whether mouse sera could neutralize two SARSr-CoVs (WIV1 and Rs3367). As expected, after three immunizations, the sera from mice immunized with CF501/RBD-Fc could potently neutralize SARSr-CoV Rs3367 and WIV1 PsV with neutralization activity (GM NT_50_: ~3000 and ~800, respectively) significantly higher than that of the Alum/RBD-Fc group (Fig. 2i, j). Significant correlations between SARS-CoV-2 RBD-binding IgG titers and NT_50_ values against SARS-CoV, WIV1, and Rs3367 were observed (Supplementary information, Fig. S3i). Furthermore, mouse sera from the CF501/RBD-Fc group showed the highest inhibitory activity against RsSHC014 S-mediated cell–cell fusion (IT_50_: 3518), with activity about 17.4- and 10.7-fold higher, respectively, than that observed in mouse sera from the Alum/RBD-Fc and cGAMP/RBD-Fc groups (Supplementary information, Fig. S3j). Thus, CF501 could enhance the ability of the RBD-Fc vaccine to elicit cross-nAbs in mice to broadly neutralize infection of divergent sarbecoviruses.

### CF501/RBD-Fc elicited robust cellular immune responses in mice

**Fig. 3:**
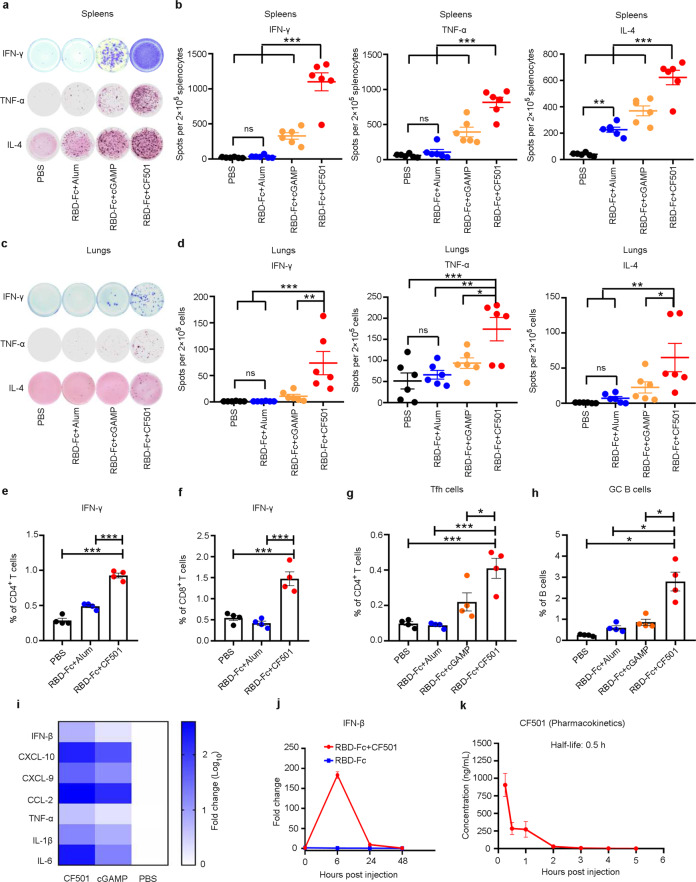
CF501/RBD-Fc induced strong T cell immune responses and transiently, but robustly, activated innate immunity. **a**–**d** SARS-CoV-2 RBD-specific T cell immune response. Mice were immunized with different vaccines. Spleens (**a** and **b**) and lungs (**c** and **d**) were collected at 7 days post-3^rd^ immunization. ELISPOT was used to determine the abundance of IFN-γ^+^, TNF-α^+^ and IL-4^+^ splenocytes (**a** and **b**) and cells in the lungs (**c** and **d**). Representative images of ELISPOT wells are shown. **e**, **f** Mice were immunized with different vaccines (CF501/RBD-Fc, Alum/RBD-Fc, and PBS control) twice. Splenocytes were stimulated with an RBD peptide pool. The proportions of IFN-γ^+^ CD4^+^ (**e**) and IFN-γ^+^ CD8^+^ (**f**) T cells were determined by intracellular cytokine staining. **g**, **h** Draining lymph nodes were collected. The percentages of Tfh cells (**g**) and GC B cells (**h**) were determined by FCS. **i** CF501 robustly activated innate immunity in draining lymph nodes. Mice were injected intramuscularly with 20 µg of CF501, 20 µg of cGAMP or an equal volume of PBS. The draining lymph nodes were collected after 6 h. The mRNA levels of cytokines and chemokines, such as IFN-β, CXCL-10, CXCL-9, CCL-2, TNF-α, IL-1β, and IL-6, were measured using RT-qPCR. **j** CF501-adjuvanted RBD-Fc transiently activated innate immunity. Mice were intramuscularly treated with 5 µg of RBD-Fc alone or formulated with 20 µg of CF501. The draining lymph nodes were collected at 0 h, 6 h, 24 h and 48 h, respectively. The mRNA level of IFN-β was measured using RT-qPCR. **k** Pharmacokinetics of CF501 following intramuscular injection in CD1 mice. CD1 mice were administrated with 75 μg CF501 and the plasmas were collected at the indicated time points. The CF501 concentrations were analyzed using LC-MS. Data are shown as means ± SEM. Statistical analyses were performed using one-way ANOVA. *P < 0.05, **P < 0.001, ***P < 0.0001.

As cellular immunity is also important for protection against virus infection, we analyzed T cell responses by using an enzyme-linked immune absorbent spot (ELISpot) assay. Splenocytes were stimulated with a peptide library that spanned the full length of the SARS-CoV-2 RBD protein. We found that CF501/RBD-Fc induced a significantly higher number of Th1-biased IFN-γ^+^ and TNF-α^+^ splenocytes than that induced by Alum/RBD-Fc or cGAMP/RBD-Fc (Fig. 3a, b). Alum/RBD-Fc induced low, or no, IFN-γ^+^ and TNF-α^+^ splenocytes, consistent with the results indicating that Alum/RBD-Fc induced much lower Th1-biased humoral immune response, as demonstrated by the limited production of IgG2a (Figs. 1e and 3a, b). Regarding Th2 immune response, the number of IL-4^+^ splenocytes induced by Alum/RBD-Fc was significantly higher than that induced by PBS. Still, the CF501/RBD-Fc-immunized mice produced an even higher number of IL-4^+^ splenocytes compared to those induced in mice immunized with either Alum/RBD-Fc or cGAMP/RBD-Fc (Fig. 3a, b). We then tested T cell response in the lungs since the lungs are the main target organ of SARS-CoV-2 infection. Strikingly, although the T cell response in the lungs was not as strong as that in the spleens, the numbers of IFN-γ^+^, TNF-α^+^, and IL-4^+^ cells in mice in the CF501/RBD-Fc group were still significantly higher than those in mice in the cGAMP/RBD-Fc and Alum/RBD-Fc groups (Fig. 3c, d). No significant difference was observed in T cell response between the Alum/RBD-Fc and PBS groups in the lungs (Fig. 3c, d). We also measured the proportions of IFN-γ^+^ CD4^+^ and IFN-γ^+^ CD8^+^ T cells. We found that CF501/RBD-Fc could induce significantly higher numbers of IFN-γ^+^ secreting CD4^+^ and CD8^+^ T cells than Alum/RBD-Fc and the PBS control (Fig. 3e, f; Supplementary information, Fig. S4a, b). Furthermore, we found that the percentages of T follicular helper cells (Tfh) and germinal center (GC) B cells within the lymph nodes in the CF501/RBD-Fc group were significantly higher than those in the Alum/RBD-Fc, cGAMP/RBD-Fc, and PBS groups (Fig. 3g, h; Supplementary information, Fig. S4c, d). Collectively, these results indicate that the STING agonist CF501 could effectively enhance the capacity of the RBD-Fc vaccine to stimulate SARS-CoV-2 RBD-specific T cell immune responses.

### CF501 robustly, but transiently, activated innate immunity with acceptable safety profile in mice

To compare the ability of CF501 and cGAMP to activate innate immunity, mice were intramuscularly administered with CF501 or cGAMP, respectively. After 6 h, we tested proinflammatory cytokines and type I IFNs in draining lymph node cells. We found that expression of cytokines, including IFN-β, CXCL-10, CXCL-9, CCL-2, TNF-α, IL-1β, and IL-6, in lymph node cells induced by CF501 was higher than that induced by cGAMP (Fig. 3i), consistent with the results from the THP-1 cells (Fig. 1c). Interestingly, CF501/RBD-Fc rapidly and robustly, but only transiently, activated innate immunity (Fig. 3j; Supplementary information, Fig. S5a). Expression of cytokines, such as IFN-β, CXCL-10, CXCL-9, CCL-2, TNF-α, IL-1β, and IL-6, in the draining lymph node cells peaked at 6 h after stimulation and subsequently diminished within 48 h (Fig. 3j; Supplementary information, Fig. S5a), thus avoiding the adverse effects potentially caused by persistent high levels of cytokine expression. Although innate immunity was activated only transiently by CF501, it should be sufficient to stimulate the required humoral and cellular immune responses. Previous studies also demonstrated that prolonged activation of innate immunity is not required for the induction of strong adaptive immunity by adjuvanted vaccines.^52,53^ We also tested the pharmacokinetic profile of CF501 in the mice following intramuscular injection of 75 μg CF501, which is more than three-fold higher than the CF501 dose (20 μg) for immunization of mice. The results revealed that the half-life of CF501 was 0.5 h and there was no detectable CF501 in the plasma after 2 h of the injection (Fig. 3k). Moreover, the levels of TNF-α, IL-6, and IL-12 in the sera of mice treated with CF501/RBD-Fc and the PBS control showed no significant difference (Supplementary information, Fig. S5b), suggesting that the innate immunity-related cytokines were transiently activated in draining lymph nodes, not in blood. Meanwhile, no significant adjuvant-related side effects were noted on mouse body weight (Supplementary information, Fig. S5c). Compared with the PBS control group, no significantly elevated level of ALT and AST in the CF501/RBD-Fc group was observed (Supplementary information, Fig. S5d). Furthermore, no significant lesion was seen in the tissues of the liver, kidney and myocardium of the mice immunized with CF501/RBD-Fc (Supplementary information, Fig. S5e). These results have implied that CF501 has the potential to be developed as a safe adjuvant for humans.

### CF501/RBD-Fc elicited the most potent immune response in rabbits

**Fig. 4:**
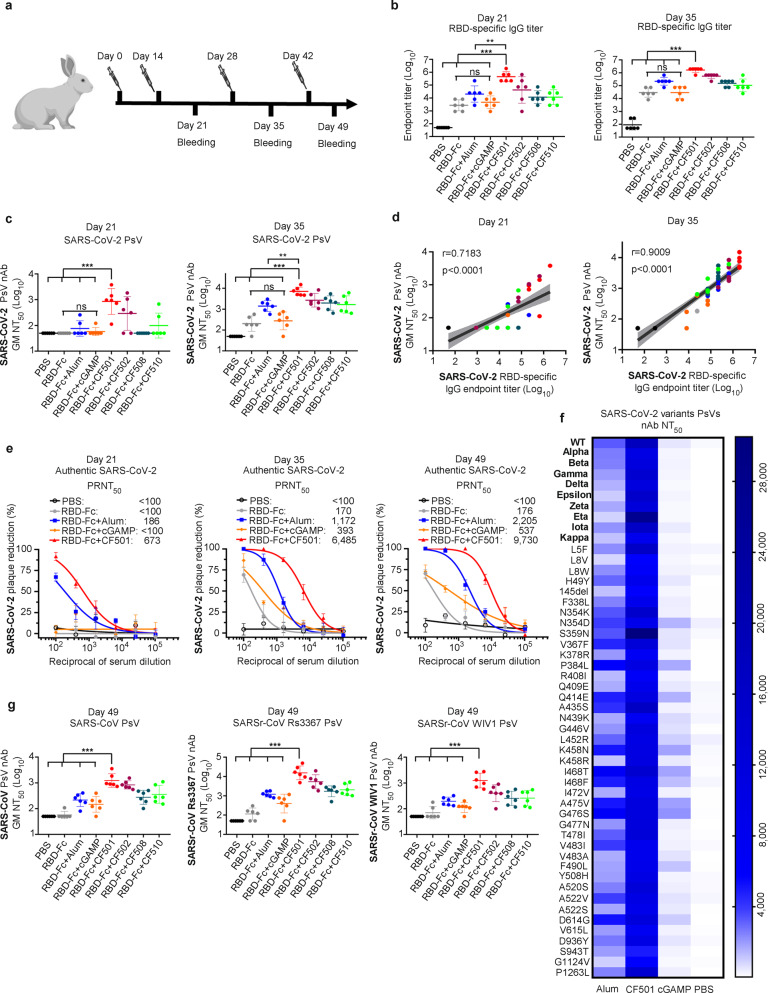
CF501/RBD-Fc induces a potent cross-neutralization immune response in rabbits. **a** Immunization procedures for rabbits. **b** SARS-CoV-2 RBD-binding endpoint titers in sera from rabbits immunized with the indicated vaccines on day 21 and day 35. Data shown are geometric means ± SD from six samples. **c** Neutralization titers against SARS-CoV-2 PsV in sera from rabbits immunized with the indicated vaccines on day 21 and day 35. Data shown are geometric means ± SD from six samples. **d** Correlation between SARS-CoV-2 RBD-binding IgG endpoint titers and NT_50_ against SARS-CoV-2 PsV in sera from rabbits on day 21 and day 35. **e** Dose-dependent inhibitory activity of pooled sera from indicated rabbit groups against authentic SARS-CoV-2 (strain: SH01) infection on days 21, 35 and 49. Data are shown as means ± SEM. **f** Neutralization titers of pooled sera from rabbits immunized with the indicated vaccines against SARS-CoV-2 variants or mutants with a single mutation PsVs. **g** Cross-neutralization activity of sera from rabbits against SARS-CoV PsV, SARSr-CoV Rs3367 PsV and SARSr-CoV WIV1 PsV on day 49. Data shown are geometric means ± SD from six samples. Statistical analyses were performed using one-way ANOVA for comparison of the neutralization antibody titers. *P < 0.05, **P < 0.001, ***P < 0.0001. Spearman rank test was used to perform the correlation analysis.

We next assessed the immune response induced by CF501/RBD-Fc in rabbits. Similar to the mouse experiment, the rabbits were randomly assigned to eight groups and immunized with PBS control, SARS-CoV-2 RBD-Fc alone or SARS-CoV-2 RBD-Fc formulated with Alum, cGAMP, CF501, CF502, CF508 or CF510, respectively. The immunization procedure is shown in Fig. 4a. The GM titers of SARS-CoV-2 RBD-specific binding antibodies elicited by the 2nd vaccination in the CF501/RBD-Fc group were about 21.5-, 96.3- and 167.5-fold higher than those in the Alum/RBD-Fc, cGAMP/RBD-Fc, and RBD-Fc alone groups, respectively (Fig. 4b; Supplementary information, Fig. S6a). Results from the SARS-CoV-2 PsV neutralization assay also showed that sera from CF501/RBD-Fc-vaccinated rabbits exhibited the most potent neutralization activity with a GM NT_50_ of 856, while GM titers in rabbits from the Alum/RBD-Fc and cGAMP/RBD-Fc groups exhibited only marginal neutralizing activity at day 21 (Fig. 4c). After three and four immunizations, CF501/RBD-Fc further elicited even higher levels of binding and nAbs titers compared with Alum/RBD-Fc and cGAMP/RBD-Fc (Fig. 4b, c; Supplementary information, Fig. S6a, b). Notably, we did not observe any increased immune response in the cGAMP/RBD-Fc or CF510/RBD-Fc groups compared to RBD-Fc alone group at days 21, 35 and 49 (Fig. 4b, c; Supplementary information, Fig. S6a, b). Unlike CF501, these results suggest that cGAMP and CF510 showed poor adjuvant effects in rabbits, but good adjuvant effects in mice. Therefore, we selected CF501, not CF510, for subsequent experiments in NHPs.

A strong correlation between RBD-binding IgG titers and NT_50_ values for neutralizing SARS-CoV-2 PsV infection on days 21, and 35, and 49 post-immunization was observed (Fig. 4d; Supplementary information, Fig. S6c). We then tested the neutralization activity of the sera from rabbits against authentic SARS-CoV-2. Consistent with the results from the SARS-CoV-2 PsV neutralization experiment, sera from rabbits immunized with CF501/RBD-Fc at days 21, 35, and 49 neutralized authentic SARS-CoV-2 with PRNT_50_ values of 673, 6485, and 9730, respectively, about 2.6-, 4.5- and 3.4-fold more potent than those from rabbits immunized with Alum/RBD-Fc and about 5.7-, 15.6- and 17.1-fold more potent than those from rabbits immunized with cGAMP/RBD-Fc (Fig. 4e). Results from the immunofluorescence assay also confirmed the potent neutralization activity of the sera from rabbits in the CF501/RBD-Fc group (Supplementary information, Fig. S6d, e). Sera from rabbits immunized with CF501/RBD-Fc could also potently inhibit SARS-CoV-2 S-mediated cell–cell fusion with an IT_50_ of 6373 (Supplementary information, Fig. S6f), about 4.3- and 38.5-fold more potent than those from rabbits immunized with Alum/RBD-Fc and cGAMP/RBD-Fc, respectively. Collectively, these results suggest that CF501 has the most potent adjuvant effect on both mice and rabbits, whereas CF510 does not work as well in rabbits.

To investigate whether the immunized rabbit serum could still neutralize these SARS-CoV-2 variants, we developed 9 pseudotyped SARS-CoV-2 variants, including Alpha (B.1.1.7), Beta (B.1.351), Gamma (P.1), Delta (B.1.617.2), Epsilon (B.1.427), Zeta (P.2), Eta (B.1.525), Iota (B.1.526), and Kappa (B.1.617.1), as well as 41 pseudotyped SARS-CoV-2 mutants with a single natural mutation in S protein. Overall, sera in the CF501/RBD-Fc group could effectively neutralize all these variants and mutants with NT_50_ values ranging from 4,481 to 30,503 (Fig. 4f). In contrast, sera from the Alum/RBD-Fc and cGAMP/RBD-Fc groups showed remarkably lower NT_50_ values in the ranges of 845–6098 and 149–2157, respectively (Fig. 4f). We next investigated the cross-neutralization activity of rabbit sera against SARS-CoV and SARSr-CoVs. CF501/RBD-Fc could elicit significantly higher nAbs in sera that effectively neutralized pseudotyped SARS-CoV, SARSr-CoV Rs3367, and SARSr-CoV WIV1, when compared to nAbs induced in sera by either cGAMP/RBD-Fc or Alum/RBD-Fc (Fig. 4g; Supplementary information, Fig. S6g). We also found a significant correlation between SARS-CoV-2 RBD-binding IgG titers and NT_50_ values against SARS-CoV, WIV1, and Rs3367 in rabbit sera collected at day 35 (Supplementary information, Fig. S6h). Moreover, CF501/RBD-Fc-elicited sera also exhibited high potency against RsSHC014 S-mediated cell–cell fusion (IT_50_: 2618), about 2.7- and 10.3-fold more potent than those from rabbits immunized with Alum/RBD-Fc and cGAMP/RBD-Fc, respectively (Supplementary information, Fig. S6i). Together, these results suggest that CF501 can enhance the capacity of the RBD-Fc vaccine to elicit highly potent cross-nAbs in rabbits against divergent sarbecoviruses, including SARS-CoV-2 and its variants and natural mutants, as well as SARS-CoV and bat SARSr-CoVs.

### Robust and durable immune responses and protection elicited by CF501/RBD-Fc in rhesus macaques

**Fig. 5:**
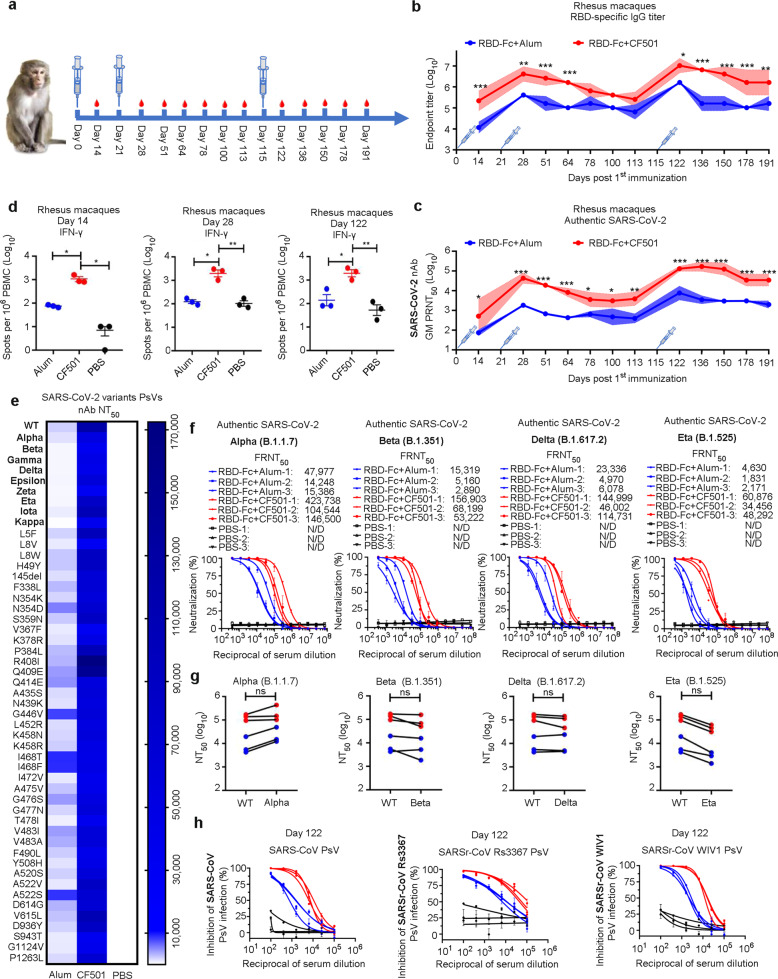
Robust and durable immune response induced by CF501/RBD-Fc in rhesus macaques. **a** Immunization and sample collection procedures for rhesus macaques. **b** SARS-CoV-2 RBD-specific binding IgG endpoint titers at the indicated time points. Data shown are geometric means ± SD. **c** Neutralization titers against authentic SARS-CoV-2 (strain: SH01) at the indicated time points using a plaque reduction assay. Data shown are the geometric means ± SD. **d** Number of IFN-γ-secreting PBMCs determined by ELISPOT after ex vivo stimulation with peptides from SARS-CoV-2 RBD on days 14, 21, and 122. **e** Neutralization antibody titers against SARS-CoV-2 VOCs and mutants with a single mutation PsVs in sera on day 28 from macaques. **f** Dose-dependent inhibitory activity of sera on day 122 from macaques against authentic SARS-CoV-2 Alpha, Beta, Delta, and Eta. N/D, not detected. **g** Comparison of the neutralization titers against SARS-CoV-2 WT strain and its variants. Red and blue dots represent macaques immunized with CF501/RBD-Fc and Alum/RBD-Fc, respectively. **h** Cross-neutralization activity of sera from macaques against SARS-CoV PsV, SARSr-CoV Rs3367 PsV and SARSr-CoV WIV1 PsV on day 122. Red line and blue line represent sera from macaques immunized with CF501/RBD-Fc or Alum/RBD-Fc, respectively. Black line represents sera from macaques treated with PBS. Data are shown as geometric means ± SD. Statistical analyses were performed using two-way ANOVA for (**b**) and (**c**). Statistical analyses for (**d**) were performed using one-way ANOVA. *P < 0.05, **P < 0.001, ***P < 0.0001.

Subsequently, we further assessed the immunogenicity of CF501/RBD-Fc in rhesus macaques. We immunized two groups (*n* = 3) of rhesus macaques three times on days 0, 21, and 115 with CF501/RBD-Fc and Alum/RBD-Fc, respectively. Three additional macaques were administered with PBS as a control group. The immunization procedure is shown in Fig. 5a. After primary immunization, we evaluated the SARS-CoV-2 RBD-binding IgG titer on day 14. CF501/RBD-Fc elicited a high titer of SARS-CoV-2 RBD-specific IgG, which was about 17.7-fold higher than that induced by Alum/RBD-Fc (Fig. 5b). Strikingly, results from the authentic SARS-CoV-2 neutralization assay indicated that sera from macaques immunized with CF501/RBD-Fc effectively neutralized authentic SARS-CoV-2 with a GM PRNT_50_ of 515 after only one immunization, which is about 6.0-fold higher than that in the Alum/RBD-Fc group (Fig. 5c; Supplementary information, Fig. S7a). After one boost, the GMT of RBD-binding antibodies in the CF501/RBD-Fc group reached 4,128,510, about 8.4-fold higher than that in the Alum/RBD-Fc group (Fig. 5b). Sera from the CF501/RBD-Fc group collected one week after the 2nd immunization (day 28 after primary immunization) were extremely potent in neutralizing authentic SARS-CoV-2 infection with a GM PRNT_50_ of 43,381, about 22.4-fold higher than that in the Alum/RBD-Fc group (Fig. 5c).

To assess the durability of the vaccine-induced immune responses, we monitored the RBD-binding and nAb titers until day 113 (92 days after the second immunization). Both binding and nAb titers of sera in CF501/RBD-Fc or Alum/RBD-Fc group gradually decreased; however, both RBD-binding and nAb titers of sera in the CF501/RBD-Fc group were always higher than those in the Alum/RBD-Fc group (Fig. 5b, c). The GM PRNT_50_ of sera in the CF501/RBD-Fc group against authentic SARS-CoV-2 virus decreased to 3902 on day 113, but it was still about 8.8-fold higher than that in the Alum/RBD-Fc group (Fig. 5c). We then performed the third immunization on day 115 and measured the titers of RBD-binding and nAbs at day 122. Very surprisingly, both RBD-binding and nAbs titers increased to exceptionally high levels in all the immunized macaques (Fig. 5b, c; Supplementary information, Fig. S7a). The GM PRNT_50_ of nAbs against authentic SARS-CoV-2 infection was 131,120, which was about 16-fold higher than that in the Alum/RBD-Fc group (Fig. 5c; Supplementary information, Fig. S7a). After the third immunization, we continuously monitored the titers of RBD-binding and nAbs in the sera for 191 days. The authentic SARS-CoV-2 nAb titer (GM PRNT_50_) in the CF501/RBD-Fc and Alum/RBD-Fc groups in the sera collected at day 191 decreased to 34,703 and 2010, respectively (Fig. 5c). Similarly, the nAb titer was closely correlated with the RBD-specific IgG titers (Supplementary information, Fig. S7b). These results suggest that CF501/RBD-Fc is able to elicit highly potent and durable nAb responses in rhesus macaques.

We then evaluated SARS-CoV-2 RBD-specific T cell responses on days 14, 28, 122, and 210 after primary immunization by IFN-γ ELISPOT assay. Peripheral blood mononuclear cells (PBMCs) were isolated from the blood of macaques and stimulated with a peptide library that spans the full length of the RBD protein. We found that CF501/RBD-Fc, but not Alum/RBD-Fc, had already induced strong IFN-γ responses by 14 days following primary immunization and these responses were maintained at similarly high levels 28 days (seven days after the 2nd immunization), 122 days (seven days after the 3rd immunization) and 210 days (95 days after the 3rd immunization) after the primary immunization (Fig. 5d; Supplementary information, Fig. S7c, d). These results suggest that CF501/RBD-Fc can also induce highly potent and durable SARS-CoV-2 RBD-specific T cell responses in rhesus macaques.

To determine whether the sera from immunized macaques could neutralize the currently circulating SARS-CoV-2 variants, we tested the neutralization activity of the sera collected at day 28 after primary immunization against 9 pseudotyped SARS-CoV-2 variants, including 4 VOCs, and 41 SARS-CoV-2 mutants with a single natural mutation in the S protein. We found that sera in the CF501/RBD-Fc group could potently neutralize all SARS-CoV-2 variants and mutants with NT_50_ values ranging from 29,584 to 172,466 (Fig. 5e). Sera in the Alum/RBD-Fc group could also neutralize all SARS-CoV-2 variants and mutants with NT_50_ values ranging from 242 to 8369 (Fig. 5e). Sera in the CF501/RBD-Fc group collected at day 122 were highly potent in neutralizing all four authentic SARS-CoV-2 variants tested, including Alpha, Beta, Delta, and Eta, with GM NT_50_ values of 186,529, 82,890, 91,469, and 46,615, respectively (Fig. 5f), while those in the Alum/RBD-Fc group exhibited lower neutralizing activity with GM NT_50_s of 21,913, 6113, 8900 and 2640 against Alpha, Beta, Delta, and Eta, respectively (Fig. 5f). We did not observe a significant difference in the neutralization activity of sera in the CF501/RBD-Fc and Alum/RBD-Fc groups against the WT strain and the Alpha, Beta, and Gamma variants, while the titer of nAbs in sera of the CF501/RBD-Fc and Alum/RBD-Fc groups against Eta variant was about 1.9-fold and 2.4-fold, respectively, lower than that against the WT strain (Fig. 5g). These results suggest that CF501/RBD-Fc is able to induce potent nAbs to combat the current circulating SARS-CoV-2 variants, including the VOCs and VOIs tested.

We next measured the cross-neutralization activity of serum antibodies against one SARS-CoV strain and two SARSr-CoVs from bats. The NT_50_s of sera in the CF501/RBD-Fc group against SARS-CoV, SARSr-CoV Rs3367, and WIV1 were higher than those in the Alum/RBD-Fc group after either one or two booster vaccinations (Fig. 5h; Supplementary information, Fig. S7e). Macaque sera in the CF501/RBD-Fc group collected on day 122 showed potent neutralizing activity against PsVs of SARS-CoV, SARSr-CoV-Rs3367, and SARSr-CoV-WIV1 with GM NT_50_ values of 7983, 66,686, and 14,849, which were about 5.6-, 6.1-, and 5.2-fold, respectively, higher than those in macaque sera of the Alum/RBD-Fc group (Fig. 5h).

Collectively, the above results suggest that the CF501-adjuvanted, Fc-conjugated RBD from the SARS-CoV-2 WT strain (CF501/RBD-Fc) can induce highly potent, broad-spectrum and durable nAb and T cell responses in NHPs against SARS-CoV-2 and its variants and mutants, as well as SARS-CoV and some bat SARSr-CoVs.

### CF501/RBD-Fc elicited long-term protective immunity in human ACE2 transgenic (hACE2-Tg) mice against SARS-CoV-2 challenge

**Fig. 6:**
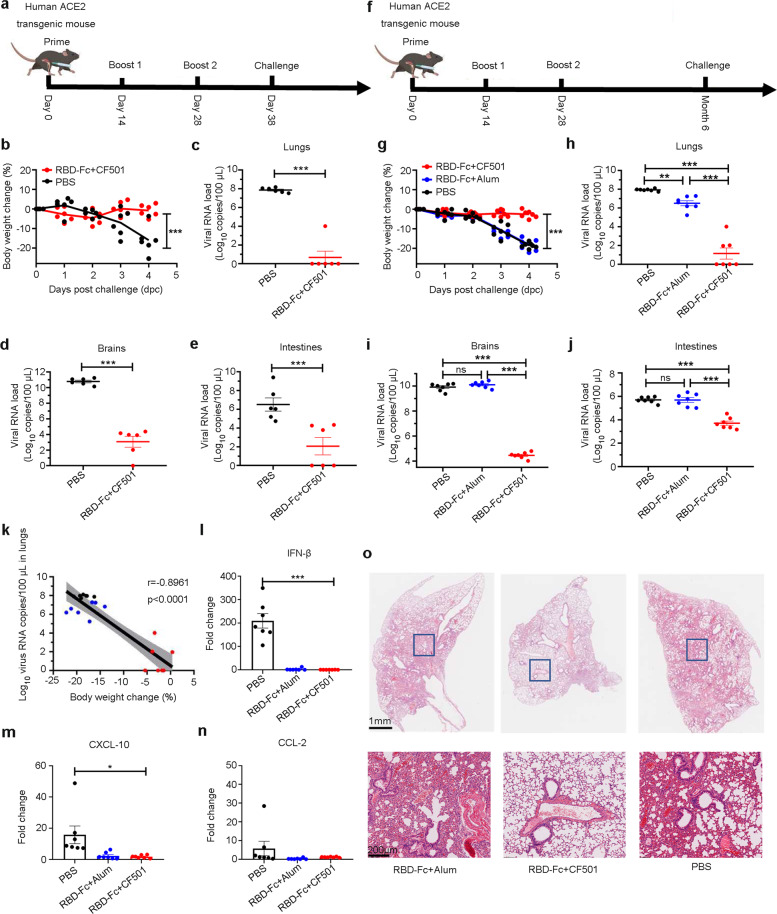
CF501/RBD-Fc provides long-term protection against SARS-CoV-2 challenge in hACE2-Tg mice. **a** Immunization and challenge procedure for the hACE2-Tg mice to evaluate immediate protection efficacy. The hACE2-Tg mice were immunized with CF501/RBD-Fc at days 0, 14, and 28, respectively. Control hACE2-Tg mice were treated with an equal volume of PBS. The SARS-CoV-2 (strain: SH01) challenge was conducted on day 38. **b** SARS-CoV-2 (strain: SH01) challenge was conducted 10 days post-final immunization. Body weight changes were monitored for hACE2-Tg mice in CF501/RBD-Fc and control groups. **c**–**e** The SARS-CoV-2 challenge (strain: SH01) was conducted 10 days post-final immunization. SARS-CoV-2 viral loads were determined using RT-qPCR at 4 dpc in lungs (**c**), brains (**d**), and intestines (**e**). Data are shown as mean ± sem. **f** Immunization and challenge procedures for evaluating the long-term protective efficacy in hACE2-Tg mice. The hACE2-Tg mice were immunized with CF501/RBD-Fc and Alum/RBD-Fc on days 0, 14, and 28, respectively. Control hACE2-Tg mice were treated with an equal volume of PBS. The SARS-CoV-2 (strain: SH01) challenge was conducted 6 months post-1^st^ immunization. **g** The SARS-CoV-2 (strain: SH01) challenge was conducted 6 months post-1^st^ immunization. Body weight changes were monitored for hACE2-Tg mice in CF501/RBD-Fc, Alum/RBD-Fc, and control groups. **h**–**j** The SARS-CoV-2 (strain: SH01) challenge was conducted 6 months post-1^st^ immunization. SARS-CoV-2 viral loads were determined using RT-qPCR at 4 dpc in lungs (**h**), brains (**i**), and intestines (**g**). Data are shown as means ± SEM. **k** Correlation between the body weight change and viral loads in lungs for mice challenged at 6 months. **l**–**n** Cytokines and chemokines, such as IFN-β (**l**), CXCL-10 (**m**), and CCL-2 (**n**), in lungs from mice challenged at 6 months post-1^st^ immunization were determined using RT-qPCR. Data were shown as means ± SEM. **o** Histopathological staining of lungs from mice challenged at 6 months. Statistical analyses were performed using two-way ANOVA for (**b**) and (**g**). Statistical analyses for (**c**), (**d**), (**e**), (**h**), (**i**), (**j**), (**l**) and (**m**) were performed using one-way ANOVA. *P < 0.05, **P < 0.001, ***P < 0.0001.

To assess the protective efficiency of CF501/RBD-Fc, six hACE2-Tg mice were immunized with CF501/RBD-Fc three times with an interval of 14 days (Fig. 6a). Six additional hACE2-Tg mice were administered with PBS as a control group. Ten days after the three immunizations, mice were challenged with 0.5 × 10^5^ PFU authentic SARS-CoV-2 (Fig. 6a). After infection, mild or no, body weight loss was observed in the CF501/RBD-Fc group, while sustained body weight loss with a maximum loss of 16% at 4 day post-challenge (dpc) was found in the viral control group (Fig. 6b). More strikingly, the average viral loads in the lungs, brains, and intestines of the hACE2-Tg mice in the PBS control group were 7.86, 10.79, and 6.51 log_10_ copies/100 μL, respectively, while those in the CF501/RBD-Fc group were about 0.67, 3.07, and 2.06 log_10_ copies/100 μL, respectively (Fig. 6c–e).

To determine whether CF501/RBD-Fc could provide long-term protection, three groups (*n* = 7) of hACE2-Tg mice were immunized with CF501/RBD-Fc, Alum/RBD-Fc, and PBS (as a control group), respectively. After two booster vaccinations, the mice were challenged with SARS-CoV-2 at six months post-1st immunization (Fig. 6f). Mice in the PBS control and Alum/RBD-Fc groups suffered severe body weight loss. The average weight loss at 4 dpc was 18.5% and 18.2% in the PBS and Alum/RBD-Fc groups, respectively, while no body weight loss was observed in mice immunized with CF501/RBD-Fc (Fig. 6g). The viral loads in the lungs of mice immunized with PBS, Alum/RBD-Fc, and CF-501/RBD-Fc were 7.93, 6.51, and 1.14 log_10_ copies/100 μL, respectively (Fig. 6h). The viral loads in brains and intestines of mice immunized with PBS, Alum/RBD-Fc and CF-501/RBD-Fc were 9.92, 10.11, and 4.46 log_10_ copies/100 μL, respectively, and 5.71, 5.69, and 3.71 log_10_ copies/100 μL, respectively (Fig. 6i, j). The viral loads in the lungs were inversely correlated with the percentage of body weight loss (Fig. 6k).

We then tested the viral subgenomic RNA (sgRNA) in these samples since sgRNA could reflect viral replication. Consistent with the results above, the sgRNA loads in the lungs of mice from the PBS control, Alum/RBD-Fc and CF501/RBD-Fc groups were 5.81, 3.72, and 2.35 log_10_ copies/100 μL, respectively (Supplementary information, Fig. S8a), while those in the brains of mice from the PBS control, Alum/RBD-Fc, and CF501/RBD-Fc groups were 8.57, 9.66, and 2.71 log_10_ copies/100 μL, respectively (Supplementary information, Fig. S8b). The viral sgRNA loads in the lungs were also inversely correlated with the percentage of body weight loss (Supplementary information, Fig. S8c).

We also evaluated the expression of proinflammatory cytokines and histopathologic changes in the lungs of hACE2-Tg mice immunized with CF501/RBD-Fc, Alum/RBD-Fc, and PBS control. The mRNA levels of IFN-β, CXCL-10, and CCL-2 in the PBS group increased about 209.2-, 15.8-, and 5.7-fold, while those in the CF501/RBD-Fc and Alum/RBD-Fc groups remained at background levels (Fig. 6l–n). Only limited histopathological changes in the lungs of the mice immunized with CF501/RBD-Fc were observed, while mice in the control and Alum/RBD-Fc groups showed typical viral interstitial pneumonia (Fig. 6o).

The above results suggest that CF501/RBD-Fc could provide long-term (at least six months in mice) protection of vaccinated hACE2-Tg mice against body weight loss and histopathological changes, as well as against viral infection and replication in the lungs, brains, and intestines.

### CF501/RBD-Fc protected rhesus macaques against SARS-CoV-2 challenge

**Fig. 7:**
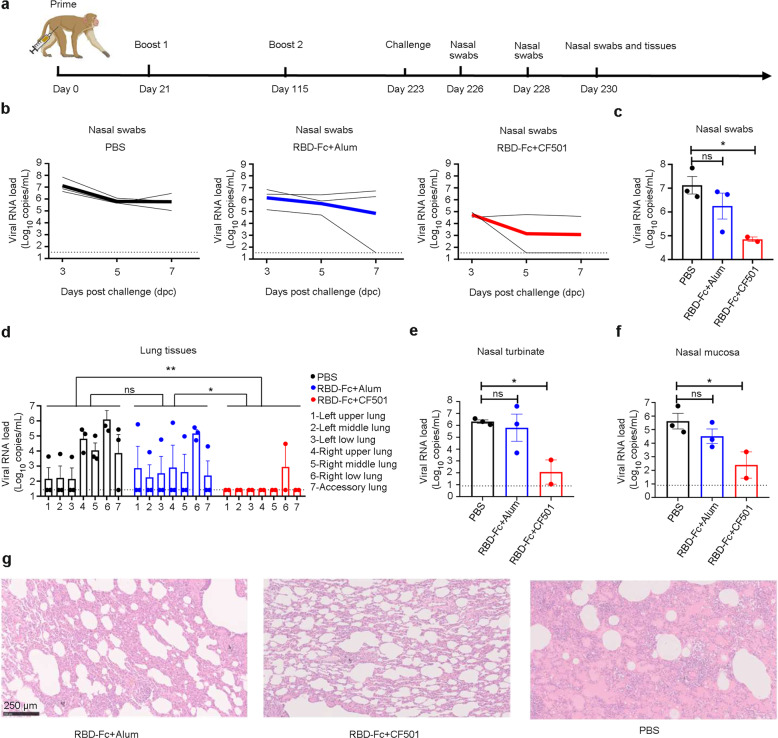
Protective efficacy of CF501/RBD-Fc in rhesus macaques. **a** Immunization and challenge procedures for rhesus macaques. Rhesus macaques were immunized with CF501/RBD-Fc and Alum/RBD-Fc on day 0, 21, and 115, respectively. The control group was treated with an equal volume of PBS. The SARS-CoV-2 (Strain: WH-09) challenge was conducted on day 223. Nasal swabs were collected at 3, 5, and 7 dpc. Macaques were sacrificed at 7 dpc. Tissues, such as lungs and nasal turbinate, were collected. **b** SARS-CoV-2 viral loads in the nasal swabs at 3, 5, and 7 dpc were determined using RT-qPCR assay. Bold lines reflect mean values. **c** Peak viral loads in nasal swabs after challenge. **d** SARS-CoV-2 viral loads in the indicated lung lobes from macaques at 7 dpc were determined using RT-qPCR assay. **e**, **f** SARS-CoV-2 viral loads in nasal turbinate (**e**) and nasal mucosa (**f**) from macaques at 7 dpc. **g** Histopathological staining of the lungs from macaques at 7 dpc. The dotted line represents the limit of detection. Statistical analyses were performed using two-way ANOVA for (**d**). Statistical analyses for (**c**), (**e**), and (**f**) were performed using one-way ANOVA. *P < 0.05, **P < 0.001, ***P < 0.0001.

To further assess the long-term protective potential of CF501/RBD-Fc, vaccinated rhesus macaques and contemporaneous control macaques were intratracheally challenged with 1 × 10^6^ TCID_50_ SARS-CoV-2 at 223 days post-1st immunization (108 days post-final immunization) (Fig. 7a). Two macaques in the CF501/RBD-Fc group were challenged since the macaque in this group with the highest nAb titer was selected to perform a nAb-related study for another project. Consistent with the results previously reported,^44^ no signs of clinical disease, including body weight and body temperature changes, were observed (Supplementary information, Fig. S9a, b). The median peak viral loads at 3 dpc in nasal swabs of macaques immunized with PBS control, Alum/RBD-Fc, and CF501/RBD-Fc were 7.12, 6.15, and 4.74 log_10_ copies/mL, respectively (Fig. 7b, c), and the median viral loads in the CF501/RBD-Fc group was about 240-, 450-, and 500-fold lower than those in the PBS group at 3, 5 and 7 dpc, respectively (Fig. 7b). At 7 dpc, the macaques were euthanized to collect the lung lobes for RT-qPCR to determine the viral loads. The viral loads in lung tissues of macaques in the CF501/RBD-Fc group were significantly lower than those in the Alum/RBD-Fc and PBS control groups (Fig. 7d). One macaque in the CF501/RBD-Fc group had no detectable viral load in any of the lung lobes. Only moderate viral loads were detected in one lung lobe of the other macaque in the CF501/RBD-Fc group (Fig. 7d). Similarly, the viral loads in both nasal turbinate and nasal mucosa of macaques in the CF501/RBD-Fc group were significantly lower than those in the Alum/RBD-Fc and PBS groups (Fig. 7e, f). We also found that viral loads in the samples of throat tonsil, cervical lymph node, rectum, and colon of macaques in the CF501/RBD-Fc group were remarkably lower than those in the Alum/RBD-Fc and PBS groups (Supplementary information, Fig. S9c–f). Severe interstitial pneumonia was observed in macaques in the PBS control group, while only moderate or mild pathological lung changes were observed in the Alum/RBD-Fc and CF501/RBD-Fc groups, respectively (Fig. 7g). Vaccine-associated enhanced respiratory disease (VAERD)^54–56^ were not observed in the vaccinated macaques. All above results suggest that CF501/RBD-Fc can induce durable protection in NHPs tested against the SARS-CoV-2 challenge.

## Discussion

Because of the urgent need to develop vaccines to combat the COVID-19 pandemic, a world history record has been achieved this year, i.e., more than a dozen COVID-19 vaccines, such as the mRNA-based vaccines of Moderna and Pfizer-BioNTech (mRNA-1273 and BNT162b2), the inactivated virus-based vaccines of Sinopharm and Sinovac (BBIBP-CorV and CoronaVac), the viral vector-based vaccine of AstraZeneca (ChAdOx1) and Janssen’s vaccine (Ad26.COV2S), have been authorized for emergency use in many countries, just 14–18 months after the identification of SARS-CoV-2 at the beginning of 2020.^23,25,57–64^ These COVID-19 vaccines provide protection against SARS-CoV-2 infection at rates ranging from 50.4 to 95%.^65,66^ However, they are less effective against SARS-CoV-2 VOCs, resulting in increasing breakthrough infection.^65^ Therefore, it is essential to develop more effective pan-sarbecoviruses vaccines against SARS-CoV-2 and its variants, as well as SARS-CoV and SARSr-CoVs, that may cause future outbreaks of sarbecovirus infection. Towards this goal, nanoparticles co-displaying multiple distinct RBDs from beta-coronaviruses could induce cross-nAbs in mice, providing a strategy for simultaneous protection against SARS-CoV-2 and some zoonotic sarbecovirus infections.^67,68^ Similarly, nanoparticles displaying the SARS-CoV-2 RBD formulated with 3M-052 and alum could induce cross-nAb responses against multiple sarbecoviruses in macaques.^69^ More recently, multiplexed-chimeric spike mRNA which contains admixtures of different RBD, NTD, and S2 from many CoVs, such as SARS-CoV-2, SARS-CoV, RsSHC014, and HKU3-1, has been reported to induce broadly nAbs against multiple high-risk sarbecoviruses in mice.^70^ These studies have promoted the development of pan-sarbecovirus vaccine. Strategies underlying the development of pan-sarbecovirus vaccines by others have mainly focused on the selection of multiple antigens from different SARS-CoV-2 VOCs. We, on the other hand, have simply used the RBD from the original SARS-CoV-2 strain and a STING agonist-based adjuvant. This novel adjuvant is expected to markedly enhance the immunogenicity of the RBD tested, allowing this STING agonist-adjuvanted RBD-Fc vaccine to elicit highly potent and, thus, durable and broad-spectrum nAbs and T cell immune responses against SARS-CoV-2 and its variants, as well as other sarbecoviruses.

To develop a pan-sarbecovirus vaccine with a capacity to induce broadly cross-nAbs against multiple sarbecoviruses, we chose RBD-Fc as an optimal immunogen because 1) RBD contains the main neutralizing epitopes, but not the predominant non-neutralizing epitopes in S protein; 2) the human IgG Fc fragment in the vaccine causes the RBD to automatically dimerize, exposing the neutralizing epitopes; and 3) RBD-Fc can bind to the Fc receptor on the antigen-presenting cells for better presentation of the vaccine’s antigen.^25,30,71–73^ To further increase the immunogenicity of RBD-Fc, an adjuvant better than Alum should be selected. We have previously reported that cGAMP, which is the natural STING agonist, is an excellent adjuvant to enhance the immunogenicity of a nanoparticle-based influenza A vaccine.^49^ However, it has several limitations when used with an intramuscularly administered vaccine, even though cGAMP is the natural STING agonist. For example, it does not easily accumulate in the cell cytoplasm because it contains multiple negative charges. cGAMP can be quickly degraded by phosphodiesterases at the inoculation site, resulting in suboptimal adjuvant effects.^51,74^ Different from cGAMP, CF501 is a small-molecule non-nucleotide STING agonist able to more easily enter the cells to activate the STING pathway, as demonstrated by the high levels of phosphorylated STING, TBK1 and IRF3 in THP-1 cells and proinflammatory cytokines and type I IFNs in draining lymph node (Figs. 1b and 3i; Supplementary information, Fig. S2a). Moreover, we found that cGAMP/RBD-Fc could only elicit strong humoral and cellular immune responses in mice, not rabbits. This could be attributed to the rapid degradation in rabbits. In contrast, irrespective of the animal model tested, mice, rabbits, or macaques, the CF501/RBD-Fc group exhibited high levels of humoral and cellular immune responses. CF501 could also potently activate innate immune response in vivo, and the response was rapid, albeit transient. Therefore, CF501 did not cause overt inflammation and side effects, indicating that it might be safe for use as an adjuvant.

In addition to cGAMP, we also systematically compared the immune response elicited by Alum/RBD-Fc or CF501/RBD-Fc. Compared with the traditional Alum/RBD-Fc vaccine, CF501/RBD-Fc could greatly increase both the nAb and T-cell immune responses, extend the durability of the protective immunity against SARS-CoV-2, and enhance the cross-nAb responses against other sarbecoviruses. Specifically, in mice, CF501 could more significantly increase Th1 immune responses than Alum, since a high titer of IgG2a was observed in the CF501/RBD-Fc group, while no IgG2a was detected in the Alum/RBD-Fc group. More Fc-mediated effector functions, including antibody-dependent cellular cytotoxicity (ADCC) and antibody-dependent cellular phagocytosis (ADCP) might be mediated by IgG2a relative to IgG1.^54,75^ In addition, consistent with the results obtained for the humoral immune response, we found that Alum/RBD-Fc induced only RBD-specific Th2 cellular immune response since we observed only T cells secreting IL-4. On the other hand, CF501/RBD-Fc induced both RBD-specific Th1 and Th2 T cell immune responses, as demonstrated by the high percentages of T cells secreting IFN-γ, TNF-α, and IL-4 in mice. This suggests that the formulation with CF501 could solve the problem of poor T cell immune response induced by the recombinant RBD-Fc protein. Results for the detection of RBD-specific IFN-γ^+^ PBMC cells in macaques further demonstrated the strong Th1 cell immune response induced by CF501. The increased Th1 immune response might avoid the risk of vaccine-associated enhanced respiratory disease (VAERD) since VAERD is commonly considered to be associated with a Th2-skewed response.^54–56^

The neutralizing activity of sera from participants immunized with some first-generation COVID-19 vaccines, such as mRNA-1273, against SARS-CoV-2 VOCs, e.g., Delta and Beta, has been reported to significantly wane.^76,77^ Here we found that CF501/RBD-Fc elicited extremely potent nAbs (GM FRNT_50_: 91,469) against the authentic Delta strain, with neutralizing potency equal to that of WT strain. Several recent studies have demonstrated that either nAb or RBD-specific antibody titer is closely correlated with vaccine-induced protective immunity in humans and NHPs.^77,78^ Here we found that nAbs in the sera of CF501/RBD-Fc-vaccinated rabbits and rhesus macaques could potently neutralize infection of the 4 authentic SARS-CoV-2 VOCs, 9 pseudotyped SARS-CoV-2 VOCs/VOIs, and 41 pseudotyped SARS-CoV-2 mutants with a single natural mutation in S protein. CF501/RBD-Fc could elicit strong protective immunity in hACE2-Tg mice and rhesus macaques against SARS-CoV-2 challenge, suggesting that a CF501/RBD-Fc vaccine candidate could induce exceptionally potent nAb and T cell responses to protect vaccinated animals from SARS-CoV-2 infection.

Durable and potent protective immunity elicited by vaccines is important to combat the long-term circulating SARS-CoV-2 and its variants. Although several COVID-19 vaccines have been demonstrated to confer protection against SARS-CoV-2 challenge in different animal models, most challenges were performed within one month after the final immunization.^24,62,79,80^ In our mouse challenge test, we demonstrated that vaccination with CF501/RBD-Fc conferred almost complete protection against SARS-CoV-2 challenge in hACE2-Tg mice even at six months post-1st immunization. As expected, the mean sgRNA in the lungs from the CF501/RBD-Fc group decreased to an undetectable level, and no body weight loss was observed in the mice (Fig. 6g; Supplementary information, Fig. S8a). In contrast, Alum/RBD-Fc was unsuccessful in achieving such effects and provided only limited protection. Although we did not specifically test how long strong protective immunity elicited by CF501/RBD-Fc would persist, the overall results did demonstrate potent protective immunity that lasted for at least six months. In addition to mice, we also found that three shots of vaccination elicited nAbs maintained at extremely high levels in macaques for as long as 191 days. The T cell response in macaques immunized with CF501/RBD-Fc also persisted for a long time since high numbers of RBD-specific IFN-γ^+^ PBMCs were detected at day 210 post-1st immunization. Moreover, the macaques immunized with CF501/RBD-Fc and then challenged exhibited significantly reduced SARS-CoV-2 infection, both in the upper and lower respiratory tracts, even at 108 days post-final immunization. This durable protective immunity might be attributed to the extremely potent immunity induced by CF501/RBD-Fc. Furthermore, early protection is extremely important to protect individuals in high-risk regions. We found that a single immunization with CF501/RBD-Fc could rapidly elicit high levels of nAbs and strong RBD-specific T cell response in macaques. However, further study to determine whether a single immunization would confer protection against the SARS-CoV-2 challenge is warranted.

Previous studies revealed that passive immunization or intramuscular DNA vaccination cannot inhibit SARS-CoV-2 infection in nasal turbinate.^81^ Moreover, several reports suggested that COVID-19 is associated with neurological and psychiatric disorders, such as anxiety or depression, even at six and 12 months post-infection, owing to the viral invasion of the central nervous system (CNS).^82–86^ The route of viral entry to the brain might also be through nasopharyngeal mucosa.^84^ In this study, we found that immune responses elicited by CF501/RBD-Fc vaccine could effectively neutralize SARS-CoV-2 infection in nasal turbinates and nasal mucosas, which is expected to play an important role in preventing viral transmission and the viral invasion of CNS. The effective inhibition of viral infection in nasal tissues might also play an important role in decreasing the mutation rate of SARS-CoV-2.

We also observed a significant increase of nAb titers in macaques after one and two boosters. Moreover, compared to that observed after the first booster, nAb titers decrease more slowly after the 2nd booster, implying that booster immunization would provide more potent and durable protective immunity. However, further studies should be performed to confirm this result using large numbers of macaques.

It was difficult to obtain enough rhesus macaques for this study since many ongoing COVID-19 vaccine projects in China need NHPs for in vivo efficacy studies. Therefore, we could only get eight rhesus macaques for the SARS-CoV-2 challenge study. Depending on the supply of rhesus macaques, we will repeat these experiments in the near future. Because of strict rules on biosafety, no authentic SARS-CoV can be tested in Biosafety Level 3 (BSL-3) facilities in China. Therefore, we used pseudotyped SARS-CoV to evaluate titers of cross-nAbs against SARS-CoV in the sera of vaccinated animals.

In summary, a pan-sarbecovirus vaccine was designed by using human IgG Fc-conjugated RBD from the original SARS-CoV-2 strain as the vaccine’s immunogen and the STING agonist CF501 as the vaccine’s adjuvant. We found that this CF501/RBD-Fc vaccine candidate could induce extremely potent, durable, and broad-spectrum nAb and T cell responses, as well protective immunity, suggesting its potential for development as a highly potent and durable pan-sarbecovirus vaccine. Because no complex manufacturing technology is needed, CF501/RBD-Fc is expected to be more easily scalable for global supply and more affordable to the people in developing countries. Most importantly, CF501/RBD-Fc vaccine is expected to be highly effective against not only the current SARS-CoV-2 variants, but also future SARS-CoV-2 variants and other emerging and reemerging sarbecoviruses that may cause outbreaks of sarbecovirus diseases.

## Materials and methods

### Cell lines

HEK293T cells, Huh-7 cells, Vero-E6 cells, and THP-1 cells were obtained from the American Type Culture Collection (ATCC). A549-ACE2 cells were obtained from Dr. Xuanming Yang at the School of Life Sciences and Biotechnology, Shanghai Jiao Tong University. Expi293F was purchased from Life Technologies. The HEK293T cells, Huh-7 cells, Vero-E6 cells, and A549-ACE2 cells were cultured and propagated in Dulbecco’s Modified Eagle’s Medium (DMEM) (Meilun, China) supplemented with 10% fetal bovine serum (FBS) (Gibco, US), streptomycin (100 mg/mL) and penicillin (100 U/mL). THP-1 cells were maintained in RPMI-1640 (Meilun) supplemented with 10% FBS (Gibco, US), streptomycin (100 mg/mL) and penicillin (100 U/mL). Expi293F was maintained in Expi293 Expression Medium.

### STING agonists

All STING agonists were synthesized in the laboratory of Fulgent Pharma LLC. All starting materials, reagents, and solvents were commercially available and the final products were characterized and analyzed with NMR, LCMS, and HPLC. The synthesis of CF501 was summarized in Supplementary information, Fig. S1. Other STING agonists CF502, CF504, CF505, CF508, CF510, and CF511 were synthesized from the appropriate starting materials in a similar fashion as that for CF501.

### Viruses

WT SARS-CoV-2 was obtained from Shanghai Medical College, Fudan University, and propagated in Vero-E6 cells. The SARS-CoV-2 variants, including Alpha (B.1.1.7), Beta (B.1.351) and Eta (B.1.525), were isolated from COVID-19 patients in Guangdong Province, China. The SARS-CoV-2 Delta (B.1.617.2) strain was provided by Guangdong Provincial Center for Disease Control and Prevention, China. All SARS-CoV-2 strains used in this study have been verified by the next-generation sequencing twice. A plaque assay was used to quantify the viral titers. Experiments related to authentic SARS-CoV-2 were conducted in the BSL-3 Laboratory of Fudan University or Guangzhou Customs District Technology Center BSL-3 Laboratory.

### Animal models

Specific-pathogen-free (SPF) female Balb/c mice (six weeks old) were purchased from Beijing Vital River Laboratory Animal Technology Co., Ltd. SPF female hACE2-Tg mice (10 weeks old) were purchased from Shanghai Model Organisms Center. CD1 mice were ordered from Charles River, Hollister, CA, male, about five weeks old. New Zealand white rabbits were purchased from Shanghai Zeyu Biological Technology Co., Ltd. Rhesus macaques were purchased from the Beijing Institute of Xieerxin Biology Resource. All animals involved in this research were in good health. All animal experiments were performed according to protocols approved by the Institutional Animal Care and Use Committee (IACUC) of the Institute of Laboratory Animal Science, Chinese Academy of Medical Sciences (DW21002), and the Institutional Laboratory Animal Care of Fudan University (20210302-083).

### Immunoblotting assay

Human monocyte THP-1 cells were treated with various STING agonist compounds at 10 μM for 3 h. After washing with PBS, the cells were lysed in 1× RIPA buffer. The protein concentration of the total cell lysate (TCL) was measured with BCA protein assay reagent. Equal amounts (30 μg) of TCL were subjected to 10% SDS-PAGE gel separation. After transfer to a PVDF membrane and blocking in TBST (TBS containing 0.1% Tween-20) containing 5% BSA at 25 °C for 1 h, the membrane was incubated with primary antibody at 4 °C overnight, followed by incubation with HRP-conjugated secondary antibody for 1 h at room temperature. PVDF membrane was washed using TBST after each antibody incubation. The specific signals were developed via ECL substrate incubation, and the images were captured using the Bio-Rad ChemiDoc machine.

### RNA isolation and RT-qPCR

Human monocyte THP-1 cells were treated with various STING agonist compounds for 5 h. After washing with PBS, the cells were lysed in TRIzol reagent. Total RNA was extracted according to the manufacturer’s instructions. The mRNA concentration was measured via a 3rd generation NanoDrop system. One microgram of mRNA was used for the reverse transcription reaction with random primers, dNTP mix, RNase inhibitor, and M-MuLV Reverse Transcriptase. SYBR green Supermix and hot-start DNA polymerase were used for qPCR, with the GAPDH gene as an internal control.

### RNA-Seq and analysis

Total RNA was extracted from THP-1 cells using TRIzol reagent (Invitrogen, Grand Island, NY) and an RNeasy Mini Kit (Qiagen, Germantown, MD). Quality of the RNA samples was determined with an Agilent 2100 Bioanalyzer. Purified RNA samples were sent to Fulgent Genetics (Temple City, CA) for library preparation and sequencing. Briefly, the library was constructed using the NEBNext® Poly(A) mRNA Magnetic Isolation Module + NEBNext® Ultra II Directional RNA Library Prep Kit (New England Biolabs, Ipswich, MA). The libraries were sequenced to a minimum depth of 60 (PE30) million reads per sample using the Illumina HiSeq4000 instrument with 150 paired-end reads.

Raw data were converted into fastq files by Illumina bcl2fastq2, v2.20. Read quality was assessed using FastQC. The sequence reads were aligned to the hg19 reference genome using STAR (version 2.6.0c). Duplicate reads, as marked by the Dragen aligner, were removed before coverage analysis. The aligned bam files were processed by HTSeq (version 0.13.5) for gene quantification followed by normalization.

Differential expression analysis was performed using DESeq2 (version 1.30.1). DESeq2 metrics were subsequently used to identify highly expressed or repressed genes for functional annotation. A total of 1380 highly expressed genes were identified by selecting genes with an adjusted *P* value less than 0.05 and a log2 fold change greater than 2. A total of 932 repressed genes were identified with an adjusted *P* value less than 0.05 and a log2 fold change less than −2. Selected genes were submitted to DAVID for functional annotation.

### Protein expression and purification

The SARS-CoV-2 RBD-Fc protein was expressed as previously described.^25^ Briefly, the expression plasmid of PcDNA3.1-RBD-Fc or PcDNA3.1-RBD-His was transfected into Expi293F cells using EZ Trans transfection reagents (Life iLAB Bio-Technology, China). The supernatants were collected after 6 days. Affinity chromatography was used to purify the RBD-Fc protein. The protein was stored at −80 °C until use.

### Animal vaccination

Forty-eight Balb/c mice were randomly assigned to 8 groups, and each group had 6 mice. PBS was used to dissolve the RBD-Fc protein and the STING agonist. The eight groups of mice were intramuscularly immunized with 5 µg of RBD-Fc alone, 5 µg of RBD-Fc formulated with an equal volume of Imject Alum adjuvant (Thermo Scientific, USA), 5 µg of RBD-Fc formulated with 20 µg cGAMP (InvivoGen, USA), 5 µg of RBD-Fc formulated with 20 µg of CF501, 5 µg of RBD-Fc formulated with 20 µg of CF502, 5 µg of RBD-Fc formulated with 20 µg of CF508, 5 µg of RBD-Fc formulated with 20 µg of CF510, and an equal volume of PBS, respectively. The mice were vaccinated three times at 14-day intervals. At one week after each immunization, serum was isolated from blood samples to analyze RBD-specific antibodies and nAbs against pseudotyped and authentic SARS-CoV-2. One week after the final immunization, mice were sacrificed, and spleens and lungs were collected to perform the ELISPOT assay.

hACE2-Tg mice were randomly assigned to five experimental groups. The first two groups were vaccinated with RBD-Fc formulated with CF501 and an equal volume of PBS, respectively, according to the immunization procedures and doses described above. At 10 days post-final immunization, the two groups of mice were challenged with SARS-CoV-2. The other three groups were immunized with RBD-Fc formulated with CF501, RBD-Fc formulated with Alum and an equal volume of PBS, respectively, using the same immunization procedures and doses. At six months post-1st immunization, the three groups of mice were challenged with SARS-CoV-2 as described above.

Forty-eight New Zealand white rabbits were assigned randomly into 8 groups, and each group had six rabbits. The eight groups of rabbits were given four intramuscular immunizations at weeks 0, 2, 4, and 6 with 10 µg of RBD-Fc alone, 10 µg of RBD-Fc formulated with an equal volume of Imject Alum adjuvant (Thermo Scientific), 10 µg of RBD-Fc formulated with 40 µg of cGAMP (InvivoGen), 10 µg of RBD-Fc formulated with 40 µg of CF501, 10 µg of RBD-Fc formulated with 40 µg of CF502, 10 µg of RBD-Fc formulated with 40 µg of CF508 and 10 µg of RBD-Fc formulated with 40 µg of CF510, respectively. Sera were collected one week after each immunization.

Nine rhesus macaques were randomly allocated to three groups, and each group had three animals. The three groups intramuscularly received 100 µg of RBD-Fc formulated with an equal volume of Imject Alum adjuvant (Thermo Scientific), 100 µg of RBD-Fc formulated 400 µg of CF501, and an equal volume of PBS, respectively. The macaques were vaccinated three times at days 0, 21, and 113, respectively. Sera were collected on days 14, 28, 51, 64, 78, 100, 122, 136, 150, 178, and 191. PBMCs were collected on days 14, 28, 122, and 210, respectively. The macaques were challenged with SARS-CoV-2 on day 223.

### Enzyme-linked immunosorbent assay (ELISA)

ELISA was used to quantify the SARS-CoV-2 or SARS-CoV RBD-specific IgG, IgG1, and IgG2a titers in serum as described previously.^25,38^ Briefly, ELISA plates were coated with 1 µg/mL RBD-His from WT SARS-CoV-2 overnight at 4 °C. The coated plates were blocked using blocking buffer (PBS containing 5% bovine serum albumin) for 2 h at 37 °C. The sera (maximum concentration, 1:100) from mice, rabbits or rhesus macaques were serially diluted 1:3 or 1:4 in PBST (PBS containing 0.05% Tween-20) for 11 dilutions in total, followed by incubation with the coated ELISA plates for 45 min at 37 °C. After washing with PBST 5 times, HRP-conjugated secondary antibody was applied to the plates and incubated for 45 min at 37 °C. HRP-conjugated goat anti-mouse IgG (Dako, Denmark), HRP-conjugated goat anti-mouse IgG1 (Thermo Scientific), and HRP-conjugated goat anti-Mouse IgG2a (Thermo Scientific) were used to detect SARS-CoV-2 RBD-specific IgG, IgG1, and IgG2a in mice. HRP-conjugated goat anti-rabbit IgG (Dako, Denmark) was used to detect SARS-CoV-2 RBD-specific IgG in rabbits. HRP-conjugated goat anti-monkey IgG (Abcam, UK) was used to detect SARS-CoV-2 RBD-specific IgG in rhesus macaques. After incubation with the secondary antibody, the plates were washed with PBST 5 times, followed by adding 3,3’,5,5’-tetramethylbenzidine (TMB) to visualize the reaction. Finally, H_2_SO_4_ was used to stop the reaction, and the data were collected at the absorbance of 450 nm using a microplate reader (Infinite M200PRO, Switzerland). ELISA endpoint titers were expressed as the highest reciprocal serum dilution exhibiting an absorbance of 450 nm > 2.1-fold over the background values. Once the titers were below 1:100, they were defined as 1:50.

### Production of the pseudotyped sarbecoviruses

Generations of the pseudotyped WT SARS-CoV-2, SARS-CoV, SARSr-CoV Rs3367, and SARSr-CoV WIV1 were conducted as previously described.^25,87,88^ Briefly, the backbone plasmid of pNL4-3.Luc.R-E- was cotransfected with one of the following plasmids into the HEK-293T cells using a transfect reagent of VigoFect (Vigorous Biotechnology, China): pcDNA3.1-SARS-CoV-2-S, or pcDNA3.1-SARS-CoV-S, or pcDNA3.1-SARSr-CoV-WIV1-S, or pcDNA3.1-SARSr-CoV-Rs3367-S. After 60 h, cell supernatant containing the pseudoviruses was harvested. Pseudotyped SARS-CoV-2 variants and mutants were constructed as previously described.^89^ Briefly, the plasmid carrying the SARS-CoV-2 spike with mutants was transfected into HEK-293T cells. Next, the G∗ΔG-VSV (VSV G pseudotyped virus) at a concentration of 7.0 × 10^4^ TCID_50_/mL was used to infect the transfected cells. After infection for 6 h, the culture supernatant was replaced with fresh DMEM containing 10% FBS after the cells were gently washed 3 times with PBS buffer containing 2% FBS. The supernatant containing the pseudotyped SARS-CoV-2 variants and mutants was then harvested and filtered (0.45-μm pore size, Millipore) at 24 h post-infection. These pseudotyped SARS-CoV-2 viruses were stored at –80 °C until use. TCID_50_ of the pseudotyped SARS-CoV-2 variants and mutants was determined by two-fold initial dilution, followed by three-fold serial dilution in a 96-well culture plate (Corning), and then 2 × 10^4^/well of Huh7 cells were seeded. After 24 h incubation, the culture supernatant was discarded to leave 100 μL in each well, and 100 μL of luciferase substrate (PerkinElmer) was added. After 2 min incubation at room temperature, 150 μL of lysate was transferred to a white solid 96-well plate for luminescence detection by a microplate luminometer (PerkinElmer, EnSite). Results were interpreted as relative luminescence unit (RLU) values. An RLU ten-fold higher than the cells-only control was considered positive. The TCID_50_ was calculated by the Reed-Muench method.

### Pseudovirus neutralization assay

Detection of the nAb against pseudotyped SARS-CoV-2 (WT), SARS-CoV, or SARSr-CoVs was conducted as previously described.^25,38,87^ Briefly, all sera from mice, rabbits, and rhesus macaques were inactivated at 56 °C for 30 min. Huh-7 cells (for SARS-CoV-2 and SARS-CoV) or A549/ACE2 (for SARSr-CoV Rs3367 or SARSr-CoV WIV1) cells at a density of 1 × 10^4^/well were seeded in a 96-well plate. Serum (maximum concentration, 1:100) was serially diluted at 1:3 or 1:4 for six dilutions in total using DMEM, followed by incubation with the pseudotyped viruses for 30 min. After that, the mixture of serum and pseudovirus was added to the Huh-7 cells. After 12 h, fresh DMEM containing 2% FBS was used to replace the cell supernatants. Cells were further cultured for 48 h before adding Promega 1× lysis buffer for 30 min. The lysate was transferred into a 96-well half-area white plate, and Firefly Luciferase Assay Kit (Promega, USA) was used to detect the luciferase activity. Luciferase values were measured using a microplate reader (Infinite M200PRO, Switzerland). Neutralization titers (NT_50_ values) were defined as the serum dilutions that could reduce 50% relative luminescence units compared to virus control wells. Once the titers were below 1:100, then the titers were considered as 1:50.

Detection of the nAb against SARS-CoV-2 variants and mutants was conducted as described above.^90^ Briefly, sera (maximal dilution: 1:100) were serially diluted (100 μL) in a 96-well plate and mixed with 50 μL (1.3 × 10^4^ TCID_50_/mL) pseudotyped SARS-CoV-2 variants and mutants. After incubation for 1 h at 37 ^o^C and 5% CO_2_, the mixture was added to 2 × 10^4^ Huh7 cells/well (100 μL). After further culture for 24 h, chemiluminescence detection was performed according to the above-mentioned titration section. NT_50_s were defined as the serum dilutions that could reduce relative luminescence units by 50% compared to the virus control wells.

### Authentic virus neutralization assay

Plaque reduction neutralization test, focus reduction neutralization test and immunofluorescence assays were used to evaluate the serum neutralization activity against authentic SARS-CoV-2 as previously described.^25,38,39^ For plaque reduction test assay, briefly, Vero-E6 cells at a density of 1 × 10^4^/well were seeded into a 96-well plate. After incubation for 24 h, serum (maximum concentration, 1:100) was serially diluted at 1:3 or 1:4 for six dilutions in total using DMEM. The diluted serum was incubated with live SARS-CoV-2 before being added to the Vero-E6 cells. After infection lasting 2 h, 1% carboxymethyl cellulose (Sigma, US) was added to the plates, and the cells were further cultured for 72 h. Finally, the cells were fixed and stained using PBS containing 4% paraformaldehyde and 1% crystal violet. After washing with water, the plaques were counted, and the PRNT_50_ was defined as the serum dilutions at which plaques were reduced by 50% compared with the viral control wells.

A focus reduction neutralization test assay was used to evaluate serum neutralization activity against SARS-CoV-2 as done previously described.^91^ Vero E6 cells were seeded into a 96-well plate one day before infection. Next day, three serially diluted Abs and SARS-CoV-2 (90-120 FFU) were combined in DMEM (2% FBS) and incubated at 37 °C for 1 h. Then a 50 μL mixture was added into a 96-well microplate seeded with Vero E6 cells. After incubation at 37 °C for 1 h, and the mixture was removed, and 100 μL MEM, containing 1.2% carboxymethylcellulose, were added. At 24 h post-infection, the overlay was discarded, and the cell monolayer was fixed with 4% paraformaldehyde solution for 2 h. After permeabilization with 0.25% Triton X-100 for 20 min at room temperature, the plates were sequentially stained with cross-reactive rabbit anti-SARS-CoV-2 N IgG (Sino Biological, Inc.) as the primary antibody and HRP-conjugated goat anti-rabbit IgG(H + L) (Jackson ImmunoResearch) as the secondary antibody at 37 °C for 1 h. The reaction was developed with KPL TrueBlue Peroxidase substrates. The numbers of SARS-CoV-2 foci were calculated using CTL ImmunoSpot S6 Ultra reader (Cellular Technology Ltd). The NT_50_ was determined by 50% focus reduction neutralization test titers.

For immunofluorescence assays, the cells treated with the mixture of live SARS-CoV-2 and serum were fixed with 4% paraformaldehyde after 48 h of infection. Rabbit anti-N antibody (Sino Biological, China) was then incubated with the cells for 1 h before adding Alexa Fluor 488-conjugated donkey anti-rabbit IgG (Thermo Fisher Scientific). Finally, the expression of SARS-CoV-2 N protein was observed by using a fluorescence microscope (Nikon Eclipse Ti-S).

### Cytokine and chemokine measurements

Three groups of C57BL/6 mice were intramuscularly injected with 20 µg of cGAMP or CF501 and an equal volume of PBS, respectively. The draining lymph nodes were harvested at 6 h post-injection and prepared for total RNA extraction with Trizol (Takara, Japan). To monitor the change of cytokine levels induced by the vaccines, two groups of mice were intramuscularly administered 5 µg of RBD-Fc alone or formulated with 20 µg of CF501, respectively. The draining lymph nodes were harvested at 6 h, 24 h, and 48 h post-injection for RNA extraction as described above. RT-qPCR was used to detect the RNA levels of the cytokines using the SYBR Green PCR kit (Takara, Japan). Glyceraldehyde 3-phosphate dehydrogenase (GAPDH) was used as an internal control. Primers for the RT-qPCR were listed in the Supplementary information, Table S1. Another two groups of mice were also injected with 5 µg RBD-Fc formulated with 20 µg CF501 and PBS, respectively. The body weights were measured, and the sera were collected for five consecutive days. Mouse TNF-α, IL-6, and IL-12 levels in serum were measured in specific ELISA kits (Thermo Scientific).

### ELISpot assay

ELISpot assays were conducted as previously described with minor modifications.^31,92^ Spleens and lungs were isolated from the immunized mice and processed into single-cell suspensions to test for IFN-γ-, TNF-α- and IL-4-positive T cells using Mouse IFN-γ ELISpotPLUS (ALP) (MabTech), Mouse TNF-α ELISpotPLUS (ALP) (MabTech) and Mouse IL-4 ELISpotPLUS (ALP) (MabTech). PBMCs were isolated from the immunized rhesus macaques to detect the IFN-γ-positive T cells using Monkey IFN-γ ELISpotPLUS (ALP) (MabTech). The protocols were conducted according to the manufacturer’s instructions. The cells were seeded into plates at a density of 200,000 cells/well and then stimulated with 2 µg/well pooled SARS-CoV-2 RBD peptide (Supplementary information, Table S2.) and 0.1 µg/mL IL-4 for 48 h. After incubation with the relevant antibodies, the antigen-specific spots were counted using an AID EliSpot Reader System (AID, Strasburg, Germany).

### Draining lymph nodes isolation and flow cytometry

The draining lymph nodes were dissected from the immunized mice and homogenized through a 40 µm strainer. ACK lysis buffer was used to remove the red blood cells. For analysis of Tfh and GC cell, the cells in the draining lymph nodes were stained at 4 °C for 30 min with a viability marker (LiveDead-FVS780, eBiosciences) and the following antibodies: anti-CD19-BV605 (1D3), anti-GL7-PE (GL7), anti-CD95-APC-R700 (JO2), anti-CD4-AF700 (RM4-5), anti-CXCR5-PE (SPRCL5) and anti-PD-1-APC (J43). FACSAria II (BD) was used to acquire the stained cells, and data were analyzed using FlowJo software (Tree Star).

### Intracellular cytokine staining

A total of 1 × 10^6^ /well of the mouse splenocytes were incubated with the SARS-CoV-2 RBD peptide pool (5 μg/mL) and anti-CD28 antibody (1 μg/mL) overnight. The cells were then incubated with Golgi-Plug (BD Pharmingen) for another 6 h and stained with anti-CD3-PE-eFluor 610 (145–2C11), anti-CD4-FITC (RM4-5), anti-CD8-APC (53–6.7), and LIVE/DEADTM dyes, respectively. After that, a fixation/permeabilization kit (BD bioscience) was used to fix and permeabilize the cells. Anti-IFN-gamma-eFluor 450 (XMG1.2) was used to stain the cells. FACSAria II (BD) was used to acquire the stained cells, and data were analyzed using FlowJo software (Tree Star).

### SARS-CoV-2 viral challenge in hACE2-Tg mice

A SARS-CoV-2 challenge test in mice was conducted as described previously.^39,93^ Briefly, immunized hACE2-Tg mice were anesthetized with pentobarbital sodium and intranasally inoculated with 0.5 × 10^5^ PFU SARS-CoV-2 diluted in 50 µL of PBS. After infection, body weights were recorded daily. Mice were euthanized at four days post-infection to dissect lungs, brains, and intestines. Trizol (Takara) was used to homogenize these organs for extraction of the total RNA. Viral loads in these samples were measured by RT-qPCR using One-Step PrimeScrip RT-PCR Kit (Takara) according to the manufacturer’s instructions. SARS-CoV-2 genomic N gene mRNA and subgenomic E gene mRNA were measured as described previously.^39,69^ The primers and probe for detection of N gene genomic mRNA were as follows: SARS-CoV-2 N-F: 5ʹ-GGGGAACTTCTCCTGCTAGAAT-3ʹ; SARS-CoV-2 N-R: 5ʹ-CAGACATTTTGCTCTCAAGCTG-3ʹ; SARS-CoV-2-N-probe: 5ʹ-FAM-TTGCTGCTGCTTGACAGATT-TAMRA-3ʹ. For detection of the E gene subgenomic RNA, the primers and probe were used as previously described.^69^ Briefly, the extracted total RNA was subjected to RT-qPCR with primers of SGMRNA-E-F: 5ʹ-CGATCTCTTGTAGATCTGTTCTC-3ʹ and SGMRNA-E-R: 5ʹ-ATATTGCAGCAGTACGCACACA-3ʹ, and probe of SGMRNA-E-Probe: 5ʹ-FAM-ACACTAGCCATCCTTACTGCGCTTCG-BHQ1-3ʹ, using the One-Step PrimeScript RT-PCR Kit (Takara). The Mastercycler ep realplex Real-time PCR System (Eppendorf) was used to perform the qPCR, and the program was as follows: 45 °C for 5 min, 95 °C for 10 s, 40 cycles of 95 °C for 10 s, 50 °C for 30 s, 72 °C for 30 s. To measure the levels of cytokines, the total RNA extracted from the lungs was reverse-transcribed using a first-strand cDNA synthesis kit (Takara, Japan). Then, qPCR was conducted using an SYBR Green PCR kit (Takara, Japan) as described above. The lungs dissected at 4 days post-infection were fixed in 4% paraformaldehyde and stained using a standard Haemotoxylin and Eosin (H&E) procedure.

### Challenge of rhesus macaques with SARS-CoV-2

Challenge of rhesus macaques with SARS-CoV-2 was conducted as previously described.^94^ Briefly, macaques immunized with CF501/RBD-Fc, Alum/RBD-Fc, or PBS control were intratracheally challenged with 1 mL of SARS-CoV-2/WH-09/human/2020/CHN at 10^6^ TCID_50_/mL. Subsequently, body weight and temperature were monitored, and nasal swabs were collected 3, 5, and 7 dpc. At 7 dpc, macaques were sacrificed, and the samples of lung lobes, nasal turbinate, and nasal mucosa were collected. Viral loads in the collected samples were detected using RT-qPCR as described above. The lung lobes were dissected, and the tissue sections were fixed in 4% paraformaldehyde and stained using a standard H&E procedure.

### Statistics

Details of statistical analysis related to each experiment are shown in the accompanying figure legends. Asterisks were used to indicate the statistical significance of differences between groups. *P < 0.005; **P < 0.001; ***P < 0.0001.

## Supplementary information

Supplementary information, Fig. S1

Supplementary information, Fig. S2

Supplementary information, Fig. S3

Supplementary information, Fig. S4

Supplementary information, Fig. S5

Supplementary information, Fig. S6

Supplementary information, Fig. S7

Supplementary information, Fig. S8

Supplementary information, Fig. S9

Supplementary information, Table S1

Supplementary information, Table S2
