# Supplementary figures and images for: A novel STING agonist-adjuvanted pan-sarbecovirus vaccine elicits potent and durable neutralizing antibody and T cell responses in mice, rabbits and NHPs
Source: Cell Res. 2022 Jan 19;32(3):269–87. doi: 10.1038/s41422-022-00612-2 (PMC8767042; doi:10.1038/s41422-022-00612-2)

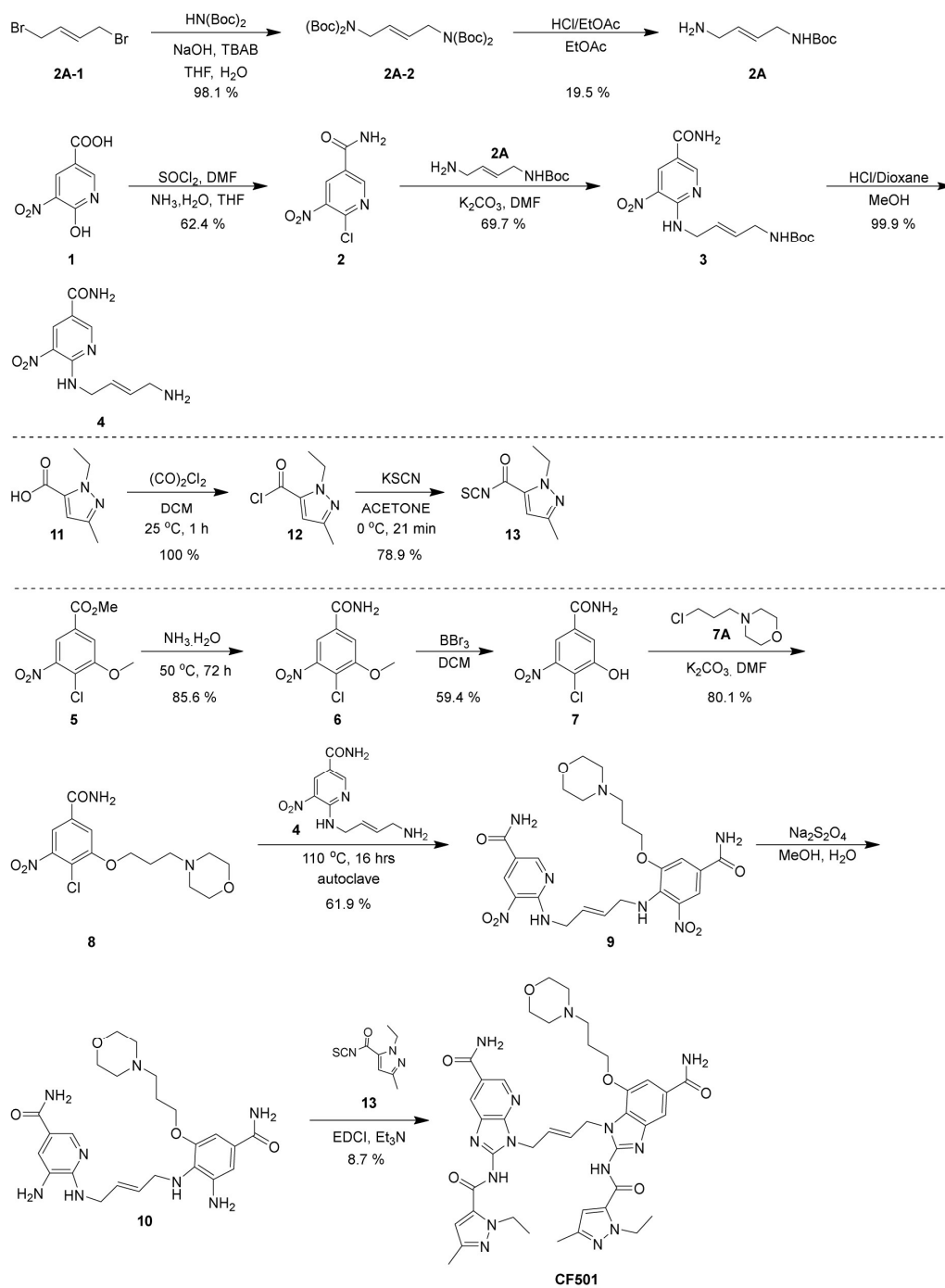

Supplementary information, Fig. S1. Synthesis of CF501

Supplement: Supplementary file 1 — Supplementary information, Fig. S1 [file 41422_2022_612_MOESM1_ESM.pdf]
