# Supplementary material for: A novel STING agonist-adjuvanted pan-sarbecovirus vaccine elicits potent and durable neutralizing antibody and T cell responses in mice, rabbits and NHPs
Source: Cell Res. 2022 Jan 19;32(3):269–87. doi: 10.1038/s41422-022-00612-2 (PMC8767042; doi:10.1038/s41422-022-00612-2)
Supplement: Supplementary file 2 — Supplementary information, Fig. S2 [file 41422_2022_612_MOESM2_ESM.pdf]

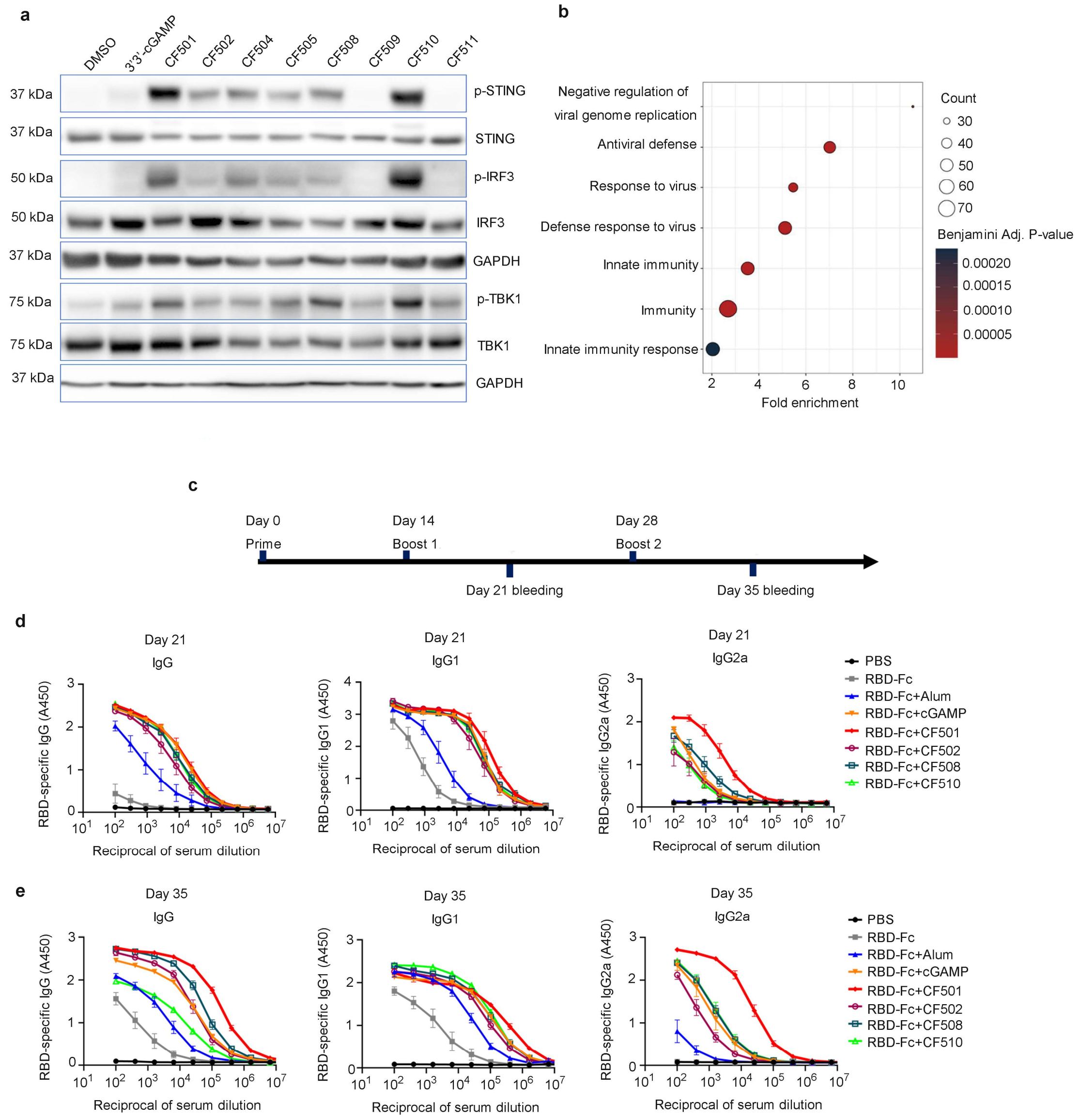

Supplementary information, Fig. S2. STING agonists rapidly activated STING in THP-1 cells and increased the binding antibody titers induced by RBD-Fc.

a THP-1 cells were treated with the indicated STING agonists for 0.5 h. Immunoblotting was used to determine the protein expression levels using the indicated antibodies.

b Genes with differential expression between CF501- and DMSO-treated THP-1 cells were identified by RNA-seq. Enrichment score scatter plot of GO, KEGG, or UniportKB terms of DAVID reported terms associated with the 1,380 highly expressed genes. The y axis includes the ontology term reported by DAVID for the differentially expressed genes ordered by the DAVID-calculated enrichment score. The x-axis

represents the enrichment score with the size of the marker scaled to the number of differentially expressed genes associated with the term. The color of the marker is scaled to the Bonferroni corrected p-value with red indicating a relatively low p-value and blue a relatively high p-value.

c Immunization procedure for mice. Balb/c mice were immunized with different vaccines on days 0, 14 and 28, respectively. Sera were isolated on day 21 and day 35.

d, e ELISA binding curves for SARS-CoV-2 RBD-specific IgG, IgG1 and IgG2a in sera on days 21(**d**) and 35 (**e**). Data are shown as the mean ± sem.
