# Supplementary material for: A novel STING agonist-adjuvanted pan-sarbecovirus vaccine elicits potent and durable neutralizing antibody and T cell responses in mice, rabbits and NHPs
Source: Cell Res. 2022 Jan 19;32(3):269–87. doi: 10.1038/s41422-022-00612-2 (PMC8767042; doi:10.1038/s41422-022-00612-2)
Supplement: Supplementary file 3 — Supplementary information, Fig. S3 [file 41422_2022_612_MOESM3_ESM.pdf]

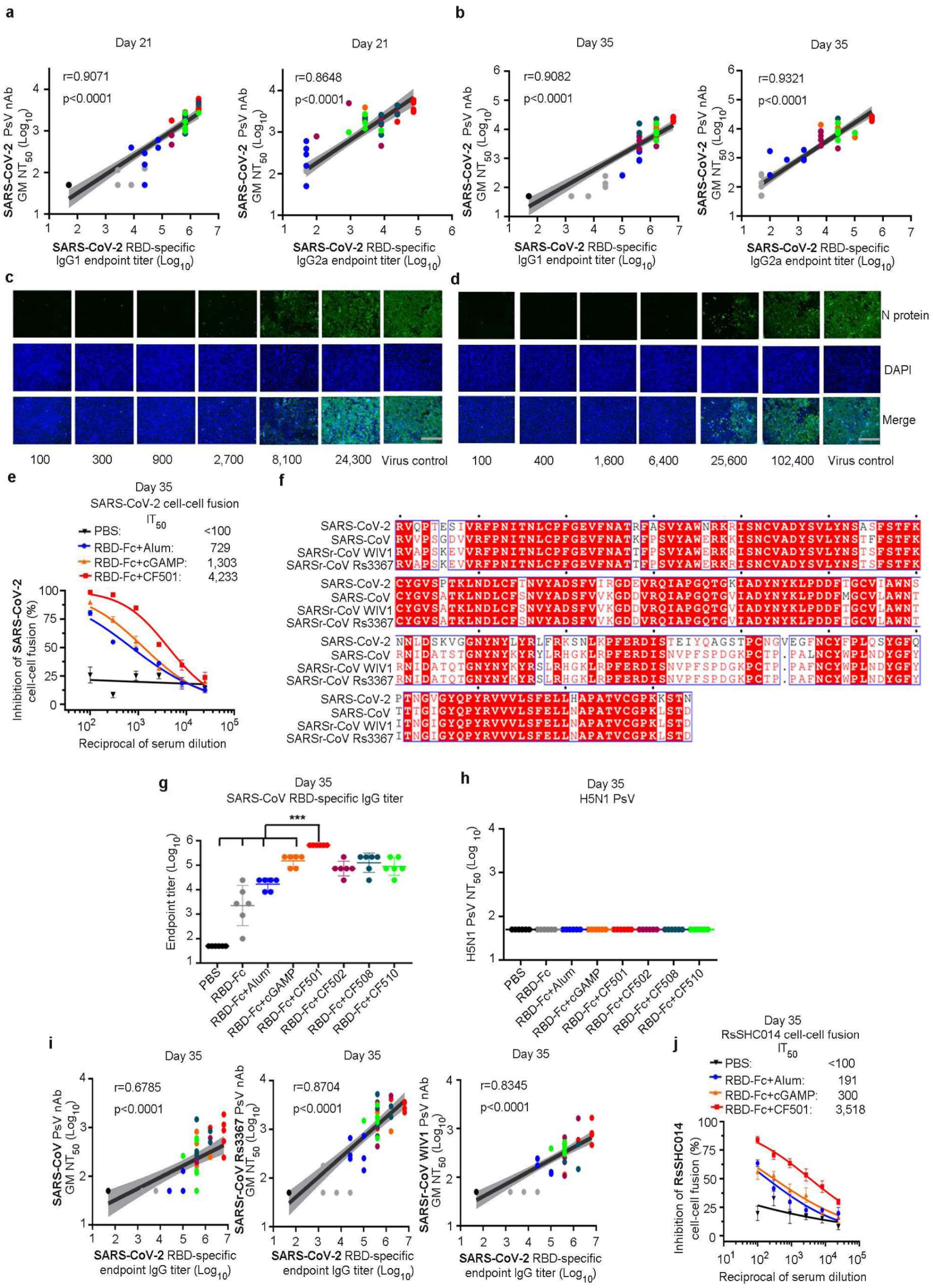

Supplementary information, Fig. S3. Robust neutralization activity induced by the CF501/RBD-Fc and the correlations in titers between RBD-binding antibodies and nAbs.

a, b Correlations between SARS-CoV-2 nAb titer and SARS-CoV-2 RBD-specific IgG1 or IgG2a endpoint titer at day 21 (**a**) and day 35 (**b**).

c, d Vero-E6 cells were infected with the SARS-CoV-2 in the presence of diluted sera

from day 21 (**c**) and day 35 (**d**). Immunofluorescence assay was used to detect SARS-CoV-2 N protein expression. Serum dilutions were indicated. Scale bars represented 400 μm.

e The inhibitory activity of sera from the indicated mouse groups at day 35 against SARS-CoV-2 S-mediated cell-cell fusion. Data are shown as mean ±sem.

f The amino acid sequence identity among SARS-CoV-2, SARS-CoV, SARSr-CoV WIV1 and SARSr-CoV Rs3367 RBD proteins.

g SARS-CoV RBD-specific IgG endpoint titers in sera from vaccinated mice.

h Neutralization activity of sera from mice immunized with RBD-Fc formulated with the indicated adjuvants against H5N1 PsV at day 35 post-immunization. Data are shown as geometric mean ± SD from six samples.

i Correlations between SARS-CoV-2 RBD-specific IgG titers and nAb titers against SARS-CoV PsV, SARSr-CoV Rs3367 PsV and SARSr-CoV WIV1 PsV.

j The inhibitory activity of sera from the indicated mouse groups at day 35 against RsSHC014 S-mediated cell-cell fusion. Data are shown as mean ±sem.

Statistical analyses were performed using one-way ANOVA for (**g**). * P<0.05, ** P <0.001, *** P <0.0001. Spearman rank test was used to perform the correlation analysis.
