# Supplementary material for: A novel STING agonist-adjuvanted pan-sarbecovirus vaccine elicits potent and durable neutralizing antibody and T cell responses in mice, rabbits and NHPs
Source: Cell Res. 2022 Jan 19;32(3):269–87. doi: 10.1038/s41422-022-00612-2 (PMC8767042; doi:10.1038/s41422-022-00612-2)
Supplement: Supplementary file 4 — Supplementary information, Fig. S4 [file 41422_2022_612_MOESM4_ESM.pdf]

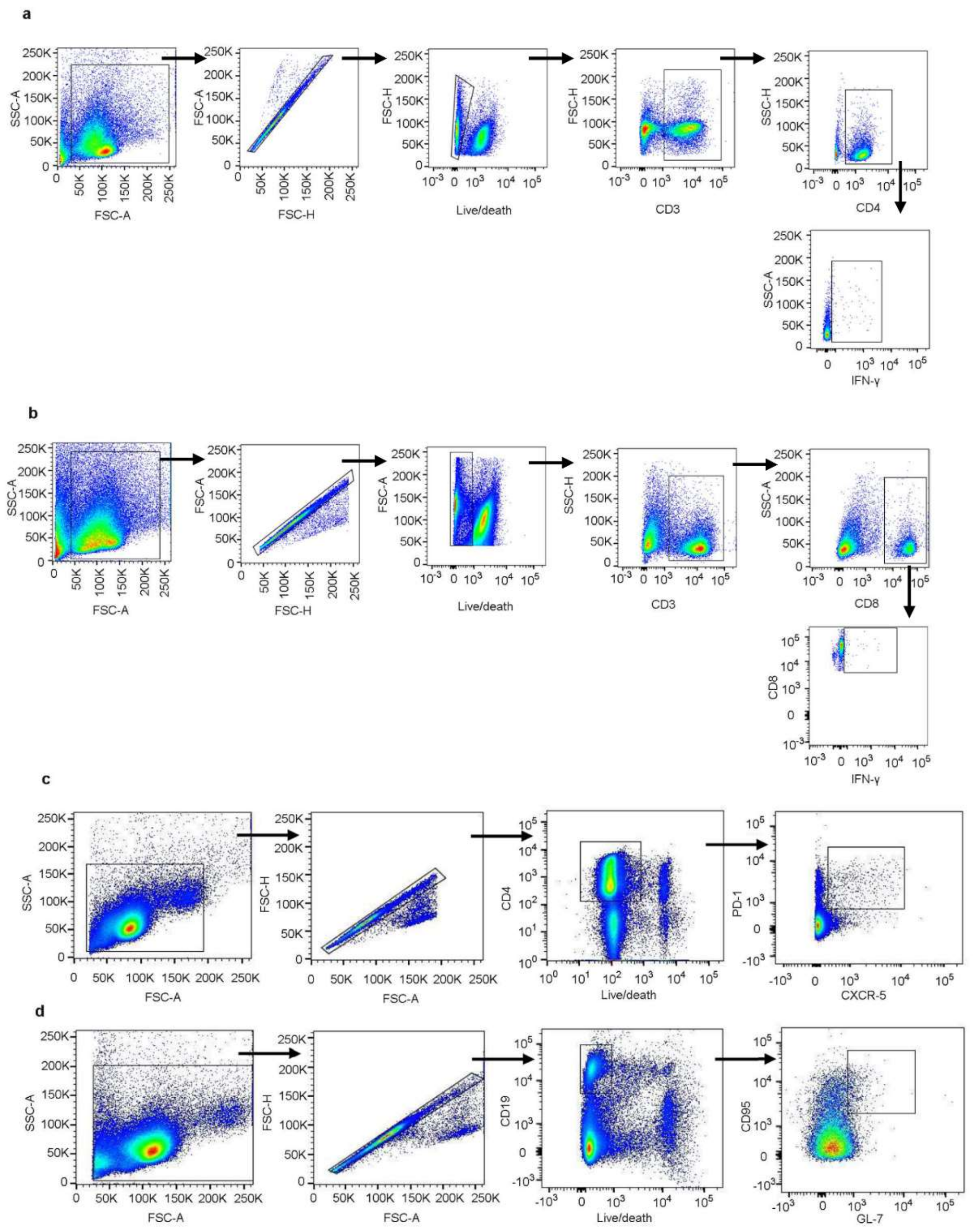

Supplementary information, Fig. S4. Representative gating strategy.

a, b Representative gating strategy for the identification of CD4^{+} IFN-γ^{+} (**a**) and CD8^{+} IFN-γ^{+} (**b**) cells in the spleens.

c, d Representative gating strategy for the identification of Tfh (**c**) and GC B (**d**) cells.
