# Supplementary material for: A novel STING agonist-adjuvanted pan-sarbecovirus vaccine elicits potent and durable neutralizing antibody and T cell responses in mice, rabbits and NHPs
Source: Cell Res. 2022 Jan 19;32(3):269–87. doi: 10.1038/s41422-022-00612-2 (PMC8767042; doi:10.1038/s41422-022-00612-2)
Supplement: Supplementary file 5 — Supplementary information, Fig. S5 [file 41422_2022_612_MOESM5_ESM.pdf]

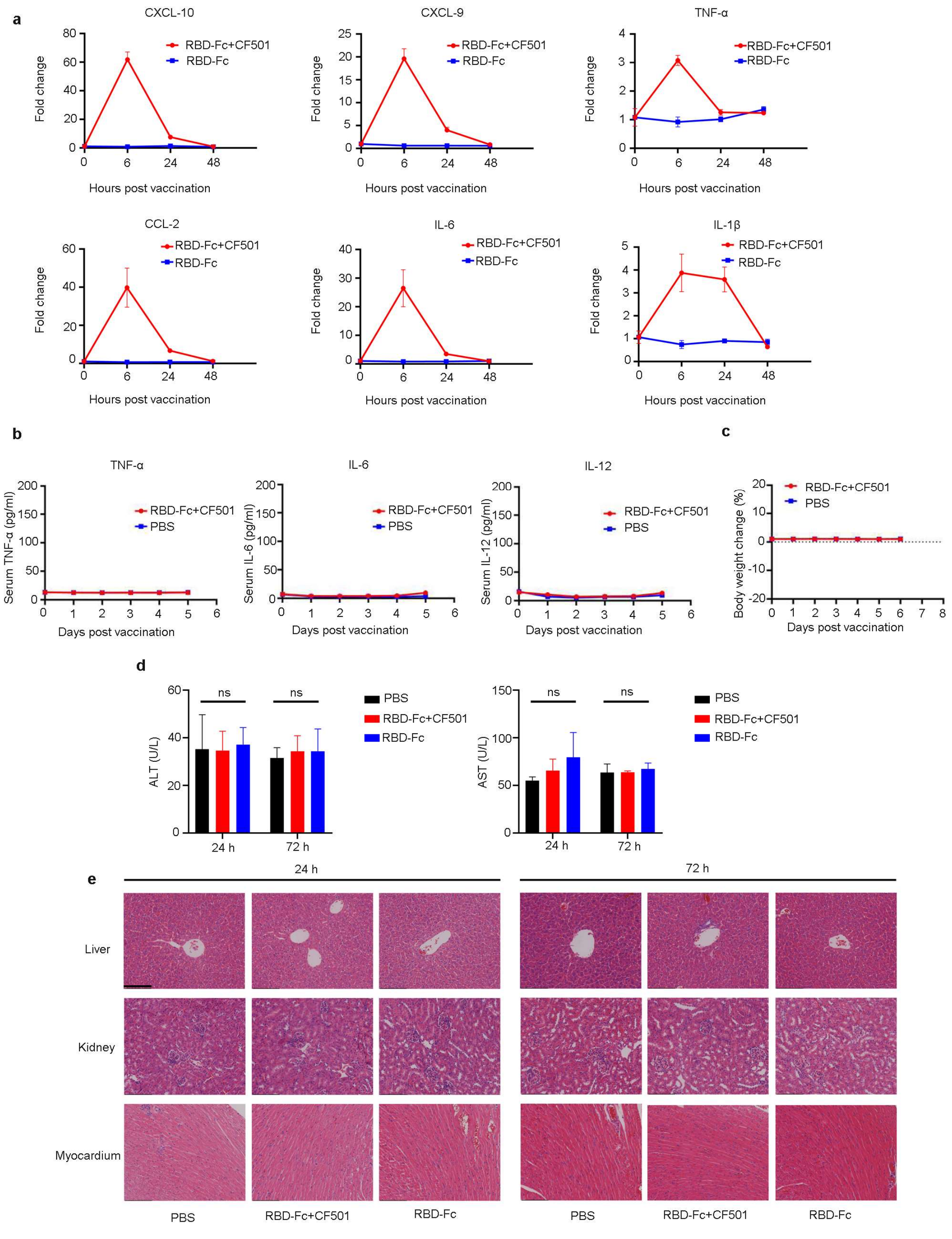

Supplementary information, Fig. S5. CF501/RBD-Fc elicits transient innate immunity with acceptable safety.

a Mice were injected intramuscularly with 5 μg of RBD-Fc alone or formulated with 20 μg of CF501. The draining lymph nodes were collected at 6 h, 24 h and 48 h. mRNA levels of CXCL-10, CXCL-9, TNF-α, CCL-2, IL-6 and IL-1β were measured by RT-qPCR.

b, c Mice were injected intramuscularly with 5 µg of RBD-Fc formulated with 20 µg CF501 or an equal volume of PBS. Sera were collected daily for 5 days. TNF-α, IL-6 and IL-12 in sera were quantified by the ELISA kits (**b**). Body weights were monitored daily for 5 days (**c**). Data are means ± sem.

d Mice were administrated intramuscularly with PBS, CF501/RBD-Fc or RBD-Fc, respectively. The sera were collected at 24 h and 72 h post injection. The ALT and AST levels were determined by an automatic biochemical analyzer.

e HE staining of the tissues in the liver, kidney and myocardium of the mice immunized intramuscularly with PBS, CF501/RBD-Fc and RBD-Fc. The indicated tissues were collected at 24 h and 72 h post-immunization. Scale bars represented 100 µm.
