# Supplementary material for: A novel STING agonist-adjuvanted pan-sarbecovirus vaccine elicits potent and durable neutralizing antibody and T cell responses in mice, rabbits and NHPs
Source: Cell Res. 2022 Jan 19;32(3):269–87. doi: 10.1038/s41422-022-00612-2 (PMC8767042; doi:10.1038/s41422-022-00612-2)
Supplement: Supplementary file 6 — Supplementary information, Fig. S6 [file 41422_2022_612_MOESM6_ESM.pdf]

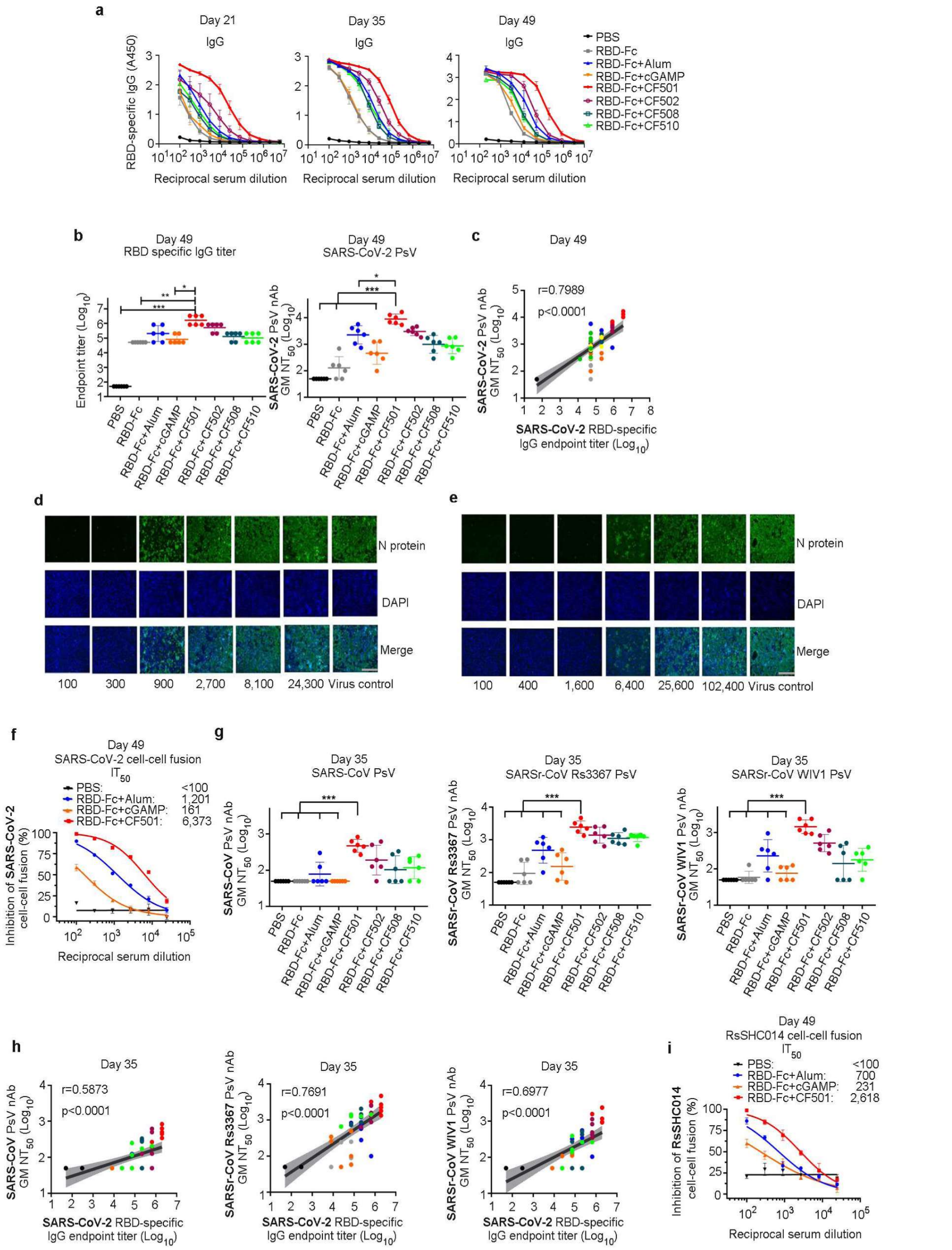

Supplementary information, Fig. S6. CF501/RBD-Fc elicited the highest cross-neutralization activity in rabbits.

a ELISA binding curve for the sera from rabbits on days 21, 35 and 49 against SARS-CoV-2 RBD. Data are shown as mean ± sem.

b SARS-CoV-2 RBD-specific IgG endpoint titers and nAb titers in sera from rabbits on day 49.

c Correlation between SARS-CoV-2 RBD-binding IgG endpoint titers and the NT50 values for neutralizing SARS-CoV-2 PsV infection in sera collected at day 49 post-immunization.

d, e Vero-E6 cells were infected with the SARS-CoV-2 in the presence of diluted sera from rabbits on day 21 (**d**) and day 35 (**e**). Immunofluorescence assay was used to detect SARS-CoV-2 N protein expression. Serum dilutions were indicated. Scale bars represented 400 μm.

f Inhibition activity of sera from the indicated rabbit groups on day 49 against SARS-CoV-2 S-mediated cell-cell fusion. Data are shown as mean ± sem.

g Cross-nAb titers against SARS-CoV PsV, SARSr-CoV Rs3367 PsV and SARSr-CoV WIV1 PsV in rabbits at day 35. Data are shown as geometric mean ± SD from six samples.

h Correlations between SARS-CoV-2 RBD-specific IgG titers and NT50s against SARS-CoV PsV, SARSr-CoV Rs3367 PsV or SARSr-CoV WIV1 PsV.

i The inhibitory activity of sera from the indicated rabbit groups on day 49 against RsSHC014 S-mediated cell-cell fusion. Data are shown as mean ± sem.

Statistical analyses were performed using one-way ANOVA for (**b**) and (**g**). * $P<0.05$, ** $P<0.001$, *** $P<0.0001$. Spearman rank test was used to perform correlation analysis.
