# Supplementary material for: A novel STING agonist-adjuvanted pan-sarbecovirus vaccine elicits potent and durable neutralizing antibody and T cell responses in mice, rabbits and NHPs
Source: Cell Res. 2022 Jan 19;32(3):269–87. doi: 10.1038/s41422-022-00612-2 (PMC8767042; doi:10.1038/s41422-022-00612-2)
Supplement: Supplementary file 7 — Supplementary information, Fig. S7 [file 41422_2022_612_MOESM7_ESM.pdf]

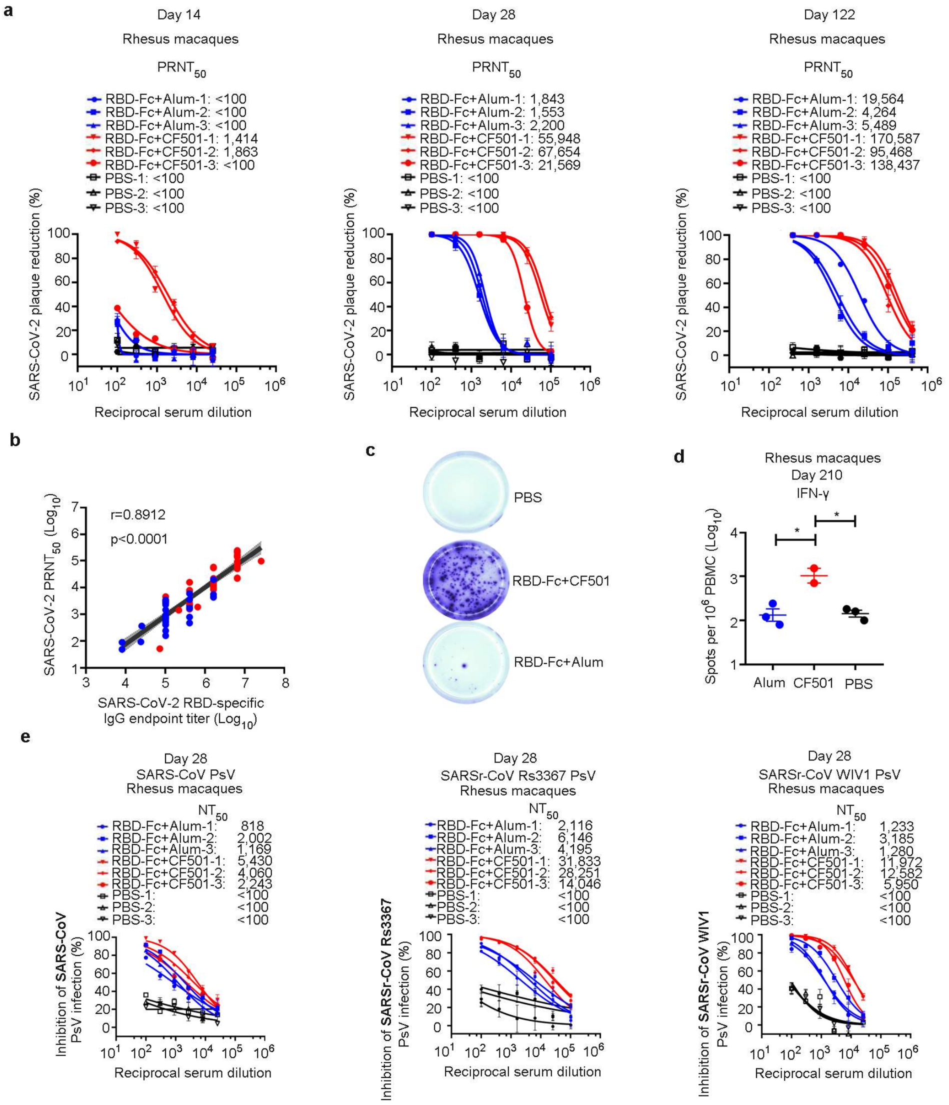

Supplementary information, Fig. S7. Robust and broad nAbs induced by CF501/RBD-Fc in rhesus macaques.

a Dose-dependent curve of inhibitory activity against authentic SARS-CoV-2 by the plaque reduction assay in Vero-E6 cells. Data are shown as mean ± sem.

b Correlation between $PRNT_{50}$ against authentic SARS-CoV-2 and ELISA binding IgG endpoint titers from day 0 to day 191.

c, d The number of IFN-γ-secreting PBMCs was determined by ELISPOT after *ex vivo* stimulation with peptides from SARS-CoV-2 RBD at day 210. Representative images of ELISPOT wells are shown (**c**).

e Cross-nAb titers against SARS-CoV PsV, SARSr-CoV Rs3367 PsV and SARSr-CoV WIV1 PsV in macaques on day 28. Data are shown as mean ± sem.

Statistical analyses were performed using one-way ANOVA for (**d**) * $P<0.05$, ** $P<0.001$, *** $P<0.0001$. Spearman rank test was used to perform correlation analysis.
