# Supplementary material for: A novel STING agonist-adjuvanted pan-sarbecovirus vaccine elicits potent and durable neutralizing antibody and T cell responses in mice, rabbits and NHPs
Source: Cell Res. 2022 Jan 19;32(3):269–87. doi: 10.1038/s41422-022-00612-2 (PMC8767042; doi:10.1038/s41422-022-00612-2)
Supplement: Supplementary file 8 — Supplementary information, Fig. S8 [file 41422_2022_612_MOESM8_ESM.pdf]

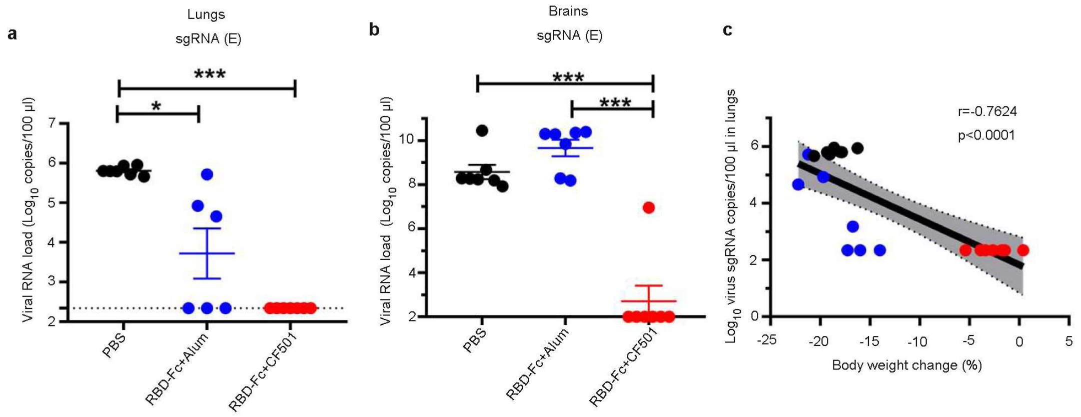

Supplementary information, Fig. S8. CF501/RBD-Fc immunized hACE2-Tg mice completely inhibited SARS-CoV-2 replication in the lungs after challenge with SARS-CoV-2 at 6 months post-1st immunization.

a, b Immunized mice were challenged with SARS-CoV-2 at 6 months post-1st immunization. Lungs and brains were collected at 7 dpc. SARS-CoV-2 sgRNA levels in lungs (**a**) and brains (**b**) were determined by RT-qPCR. Data are mean ± sem. Statistical analyses were performed using one-way ANOVA. *P<0.05, ** P <0.001, *** P <0.0001.

c Correlation between body weight change and sgRNA copies in the lungs. Spearman rank test was used to perform correlation analysis.
