# Supplementary material for: A novel STING agonist-adjuvanted pan-sarbecovirus vaccine elicits potent and durable neutralizing antibody and T cell responses in mice, rabbits and NHPs
Source: Cell Res. 2022 Jan 19;32(3):269–87. doi: 10.1038/s41422-022-00612-2 (PMC8767042; doi:10.1038/s41422-022-00612-2)
Supplement: Supplementary file 9 — Supplementary information, Fig. S9 [file 41422_2022_612_MOESM9_ESM.pdf]

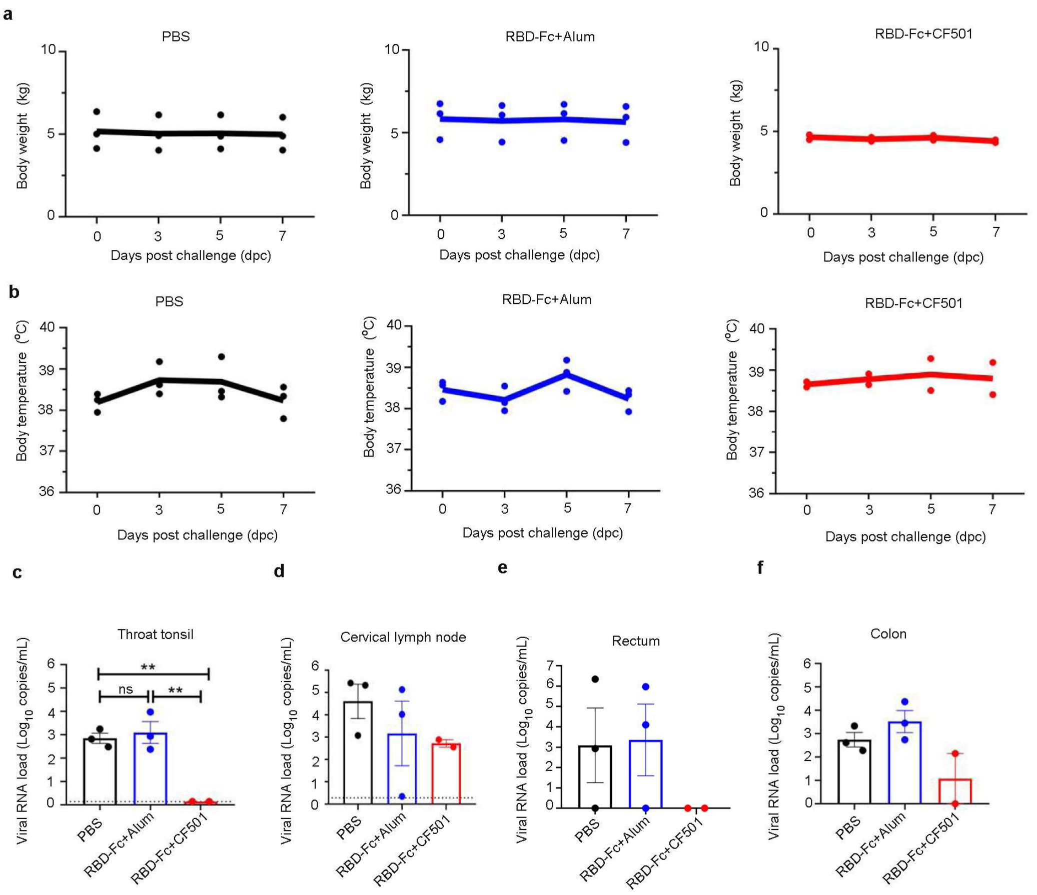

Supplementary information, Fig. S9. Clinical parameters of rhesus macaques post-SARS-CoV-2 challenge.

a Body weight of rhesus macaques after challenge.

b Body temperature of rhesus macaques after challenge.

c-f Immunized macaques were challenged with SARS-CoV-2. Throat tonsils, cervical lymph nodes, rectums and colons were collected at 7 dpc. RT-qPCR was used to quantify SARS-CoV-2 viral load in the throat tonsils (**c**), cervical lymph nodes (**d**), rectums (**e**) and colons (**f**). Data are shown as mean ± sem. Statistical analyses were performed using one-way ANOVA. * $P<0.05$, ** $P<0.001$, *** $P<0.0001$.
