# Supplementary material for: A novel STING agonist-adjuvanted pan-sarbecovirus vaccine elicits potent and durable neutralizing antibody and T cell responses in mice, rabbits and NHPs
Source: Cell Res. 2022 Jan 19;32(3):269–87. doi: 10.1038/s41422-022-00612-2 (PMC8767042; doi:10.1038/s41422-022-00612-2)
Supplement: Supplementary file 10 — Supplementary information, Table S1 [file 41422_2022_612_MOESM10_ESM.pdf]

Supplementary information, Table S1. Primers for Real-time PCR

Gene	Forward 5'-3'	Reverse 5'-3'
Human IFN-β	CTCAAGGACAGGAGGAACTTTG	CCAGTGCTCGATGAATCTTGT
Human IL6	CCAGGAGAAGATTCCAAAGATGTA	CGTCGAGGATGTACCGAATTT
Human CXCL10	CCATTCTGATTTGCTGCCTTATC	TACTAATGCTGATGCAGGTACAG
Human TNF-α	CCAGGGACCTCTCTCTAATCA	TCAGCTTGAGGGTTTGCTAC
Human ISG15	CTGAACATCCTGGTGAGGAATAA	CGAAGGTCAGCCAGAACAG
Human CCL5	TGCCCACATCAAGGAGTATTT	GATGTACTCCCGAACCCATTT
Mouse IFN-β	AGCTCCAAGAAAGGACGAACA	GCCCTGTAGGTGAGGTTGAT
Mouse CXCL10	CCAAGTGCTGCCGTCATTTTC	TCCCTATGGCCCTCATTCTCA
Mouse TNF-α	CCTGTAGCCCACGTCGTAG	GGGAGTAGACAAGGTACAACCC
Mouse CXCL9	GGAGTTCGAGGAACCCTAGTG	GGGATTTGTAGTGGATCGTGC
Mouse IL6	TAGTCCTTCCTACCCCAATTTCC	TTGGTCCTTAGCCACTCCTTC
Mouse CCL2	GGCTCAGCCAGATGCAGTTAA	CCTACTCATTGGGATCATCTTGCT
Mouse IL1β	ACATCAGCACCTCACAAGCA	TTAGAAACAGTCCAGCCCATA
