# Supplementary material for: A novel STING agonist-adjuvanted pan-sarbecovirus vaccine elicits potent and durable neutralizing antibody and T cell responses in mice, rabbits and NHPs
Source: Cell Res. 2022 Jan 19;32(3):269–87. doi: 10.1038/s41422-022-00612-2 (PMC8767042; doi:10.1038/s41422-022-00612-2)
Supplement: Supplementary file 11 — Supplementary information, Table S2 [file 41422_2022_612_MOESM11_ESM.pdf]

Supplementary information, Table S2 protein peptide pools used for ELISPOT assay.

Sequences	Protein
QTSNFRVQPTESIVRFPNIT	RBD
ESIVRFPNITNLCPFGEVFN	RBD
NLCPFGEVFNATRFASVYAW	RBD
ATRFASVYAWNRKRISNCVA	RBD
NRKRISNCVADYSVLYNSAS	RBD
DYSVLYNSASFSTFKCYGVS	RBD
FSTFKCYGVSPTKLNDLCFT	RBD
PTKLNDLCFTNVYADSFVIR	RBD
NVYADSFVIRGDEVRQIAPG	RBD
GDEVRQIAPGQTGKIADYNY	RBD
QTGKIADYNYKLPDDFTGCV	RBD
KLPDDFTGCVIAWNSNNLDS	RBD
IAWNSNNLDSKVGGNYNYLY	RBD
KVGGNYNYLYRLFRKSNLKP	RBD
RLFRKSNLKPFERDISTEIY	RBD
FERDISTEIYQAGSTPCNGV	RBD
QAGSTPCNGVEGFNCYFPLQ	RBD
EGFNCYFPLQSYGFQPTNGV	RBD
SYGFQPTNGVGYQPYRVVVL	RBD
GYQPYRVVVLSFELLHAPAT	RBD
SFELLHAPATVCGPKKSTNL	RBD
VCGPKKSTNLVKNKCVNFNF	RBD
